# Supplementary material for: Zooming into the Dark Side of Human Annexin-S100 Complexes: Dynamic Alliance of Flexible Partners
Source: Int J Mol Sci. 2020 Aug 16;21(16):5879. doi: 10.3390/ijms21165879 (PMC7461550; doi:10.3390/ijms21165879)
Supplement: Supplementary file 1 [file ijms-21-05879-s001.pdf]

Supplementary materials

Zooming into the Dark Side of Human Annexin-S100 Complexes: Dynamic Alliance of Flexible Partners

Judith Weisz,[1] and Vladimir N. Uversky [2,3,*]

[1] Departments of Gynecology and Pathology, Pennsylvania State University College of Medicine, Hershey, PA 17033, USA; E-Mail: jxw7@psu.edu

[2] Institute for Biological Instrumentation of the Russian Academy of Sciences, Federal Research Center "Pushchino Scientific Center for Biological Research of the Russian Academy of Sciences", Pushchino, 142290 Moscow, Russia

[3] Department of Molecular Medicine and USF Health Byrd Alzheimer's Research Institute, Morsani College of Medicine, University of South Florida, Tampa, FL 33612, USA

* Correspondence: vuversky@usf.edu; Tel.: +1-813-974-5816 (ext. 123); Fax: +1-813-974-7357.

Amino acid sequences of Human Annexins

>sp|P04083|ANXA1_HUMAN Annexin A1 OS=Homo sapiens OX=9606 GN=ANXA1 PE=1 SV=2
MAMVSEFLKQAWFIENEEQEYVQTVKSSKGGPGSAVSPYPTFNPSSDVAALHKAIMVKGV
DEATIIDILTKRNNAQRQQIKAAYLQETGKPLDETLKKALTGHLEEVVLALLKTPAQFDA
DELRAAMKGLGTDEDTLIEILASRTNKEIRDINRVYREELKRDLAKDITSDTSGDFRNAL
LSLAKGDRSEDFGVNEDLADSDARALYEAGERRKGTDVNVFNTILTTRSYPQLRRVFQKY
TKYSKHDMNKVLDLELKGDIEKCLTAIVKCATSKPAFFAEKLHQAMKGVGTRHKALIRIM
VSRSEIDMNDIKAFYQKMYGISLCQAILDETKGDYEKILVALCGGN
PDB ID: 1AIN; 346 residues

>sp|P07355|ANXA2_HUMAN Annexin A2 OS=Homo sapiens OX=9606 GN=ANXA2 PE=1 SV=2
MSTVHEILCKLSLEGDHSTPPSAYGSVKAYTNFDAERDALNIETAIKTKGVDEVTIVNIL
TNRSNAQRQDIAFAYQRRTKKELASALKSALSGHLETVILGLLKTPAQYDASELKASMKG
LGTDEDSLIEIICSRTNQELQEINRVYKEMYKTDLEKDIISDTSGDFRKLMVALAKGRRA
EDGSVIDYELIDQDARDLYDAGVKRKGTDVPKWISIMTERSVPHLQKVFDRYKSYSPYDM
LESIRKEVKGDLENAFLNLVQCIQNKPLYFADRLYDSMKGKGTRDKVLIRIMVSRSEVDM
LKIRSEFKRKYGKSLYYYIQQDTKGDYQKALLYLCGGDD
PDB ID: 1W7B; 339 residues

>sp|P12429|ANXA3_HUMAN Annexin A3 OS=Homo sapiens OX=9606 GN=ANXA3 PE=1 SV=3
MASIWVGHRGTVRDYPDFSPSVDAEAIQKAIRGIGTDEKMLISILTERSNAQRQLIVKEY
QAAYGKELKDDLKGDLSGHFEHLMVALVTPPAVFDAKQLKKSMKGAGTNEDALIEILTTR
TSRQMKDISQAYYTVYKKSLGDDISSETSGDFRKALLTLADGRRDESLKVDEHLAKQDAQ
ILYKAGENRWGTDEDKFTEILCLRSFPQLKLTFDEYRNISQKDIVDSIKGELSGHFEDLL
LAIVNCVRNTPAFLAERLHRALKGIGTDEFTLNRIMVSRSEIDLLDIRTEFKKHYGYSLY
SAIKSDTSGDYEITLLKICGGDD
PDB ID: 1AXN; 323 residues

>sp|P09525|ANXA4_HUMAN Annexin A4 OS=Homo sapiens OX=9606 GN=ANXA4 PE=1 SV=4
MATKGGTVKAASGFNAMEDAQTLRKAMKGLGTDEDAIISVLAYRNTAQRQEIRTAYKSTI
GRDLIDDLKSELSGNFEQVIVGMMTPTVLYDVQELRRAMKGAGTDEGCLIEILASRTPEE
IRRISQTYQQQYGRSLEDDIRSDTSFMFQRVLVSLSAGGRDEGNYLDDALVRQDAQDLYE
AGEKKWGTDEVKFLTVLCSRNRNHLLHVFDEYKRISQKDIEQSIKSETSGSFEDALLAIV
KCMRNKSAYFAEKLYKSMKGLGTDDNTLIRVMVSRAEIDMLDIRAHFKRLYGKSLYSFIK
GDTSGDYRKVLLVLCGGDD
PDB ID: 2ZOC; 319 residues

>sp|P08758|ANXA5_HUMAN Annexin A5 OS=Homo sapiens OX=9606 GN=ANXA5 PE=1 SV=2
MAQVLRGTVTDFPGFDERADAETLRKAMKGLGTDEESILTLLTSRSNAQRQEISAAFKTL
FGRDLLDDLKSELTGKFEKLIVALMKPSRLYDAYELKHALKGAGTNEKVLTEIIASRTPE
ELRAIKQVYEEEYGSSLEDDVVGDTSGYYQRMLVVLLQANRDPDAGIDEAQVEQDAQALF
QAGELKWGTDEEKFITIFGTRSVSHLRKVFDKYMTISGFQIEETIDRETSGNLEQLLLAV
VKSIRSIPAYLAETLYYAMKGAGTDDHTLIRVMVSRSEIDLFNIRKEFRKNFATSLYSMI
KGDTSGDYKKALLLLCGEDD
PDB ID: 1AVR; 320 residues

>sp|P08133|ANXA6_HUMAN Annexin A6 OS=Homo sapiens OX=9606 GN=ANXA6 PE=1 SV=3
MAKPAQGAKYRGSIHDFPGFDPNQDAEALYTAMKGFGSDKEAILDIITSRSNRQRQEVCQ
SYKSLYGKDLIADLKYELTGKFERLIVGLMRPPAYCDAKEIKDAISGIGTDEKCLIEILA
SRTNEQMHQLVAAYKDAYERDLEADIIGDTSGHFQKMLVVLLQGTREEDDVVSEDLVQQD
VQDLYEAGELKWGTDEAQFIYILGNRSKQHLRLVFDEYLKTTGKPIEASIRGELSGDFEK
LMLAVVKCIRSTPEYFAERLFKAMKGLGTRDNTLIRIMVSRSELDMLDIREIFRTKYEKS
LYSMIKNDTSGEYKKTLLKLSGGDDDAAGQFFPEAAQVAYQMWELSAVARVELKGTVRPA
NDFNPDADAKALRKAMKGLGTDEDTIIDIITHRSNVQRQQIRQTFKSHFGRDLMTDLKSE
ISGDLARLILGLMMPPAHYDAKQLKKAMEGAGTDEKALIEILATRTNAEIRAINEAYKED

YHKSLEDALSSDTSGHFRRILISLATGHREEGGENLDQAREDAQVAAEILEIADTPSGDK
TSLETRFMTILCTRSYPHLRRVFQEFIKMTNYDVEHTIKKEMSGDVRDAFVAIVQSVKNK
PLFFADKLYKSMKGAGTDEKTLTRIMVSRSEIDLLNIRREFIEKYDKSLHQAIEGDTSGD
FLKALLALCGGED
PDB ID: 1M9I; 673 residues

>sp|P20073|ANXA7_HUMAN Annexin A7 OS=Homo sapiens OX=9606 GN=ANXA7 PE=1 SV=3
MSYPGYPPTGYPPFPGYPPAGQESSFPPSGQYPYPSGFPPMGGGAYPQVPSSGYPGAGGY
PAPGGYPAPGGYPGAPQPGGAPSYPGVPPGQGFGVPPGGAGFSGYPQPPSQSYGGGPAQV
PLPGGFPGGQMPSQYPGGQPTYPSQINTDSFSSYPVFSPVSLDYSSEPATVTQVTQGTIR
PAANFDAIRDAEILRKAMKGFGTDEQAIVDVVANRSNDQRQKIKAAFKTSYGKDLIKDLK
SELSGNMEELILALFMPPTYYDAWSLRKAMQGAGTQERVLIEILCTRTNQEIREIVRCYQ
SEFGRDLEKDIRSDTSGHFERLLVSMCQGNRDENQSINHQMAQEDAQRLYQAGEGRLGTD
ESCFNMILATRSFPQLRATMEAYSRMANRDLLSSVSREFSGYVESGLKTILQCALNRPAF
FAERLYYAMKGAGTDDSTLVRIVVTRSEIDLVQIKQMFAQMYQKTLGTMIAGDTSGDYRR
LLLAIVGQ
No structure; 488 residues

>sp|P13928|ANXA8_HUMAN Annexin A8 OS=Homo sapiens OX=9606 GN=ANXA8 PE=1 SV=3
MAWWKSWIEQEGVTVKSSSHFNPDPDAETLYKAMKGIGTNEQAIIDVLTKRSNTQRQQIA
KSFKAQFGKDLTETLKSELSGKFERLIVALMYPPYRYEAKELHDAMKGLGTKEGVIIEIL
ASRTKNQLREIMKAYEEDYGSSLEEDIQADTSGYLERILVCLLQGSRDDVSSFVDPGLAL
QDAQDLYAAGEKIRGTDEMKFITILCTRSATHLLRVFEEYEKIANKSIEDSIKSETHGSL
EEAMLTVVKCTQNLHSYFAERLYYAMKGAGTRDGTLIRNIVSRSEIDLNLIKCHFKKMYG
KTLSSMIMEDTSGDYKNALLSLVGSDP
PDB ID: 1W3W; 327 residues

>sp|O76027|ANXA9_HUMAN Annexin A9 OS=Homo sapiens OX=9606 GN=ANXA9 PE=1 SV=3
MSVTGGKMAPSLTQEILSHLGLASKTAAWGTLGTLRTFLNFSVDKDAQRLLRAITGQGVD
RSAIVDVLTNRSREQRQLISRNFQERTQQDLMKSLQAALSGNLERIVMALLQPTAQFDAQ
ELRTALKASDSAVDVAIEILATRTPPQLQECLAVYKHNFQVEAVDDITSETSGILQDLLL
ALAKGGRDSYSGIIDYNLAEQDVQALQRAEGPSREETWVPVFTQRNPEHLIRVFDQYQRS
TGQELEEAVQNRFHGDAQVALLGLASVIKNTPLYFADKLHQALQETEPNYQVLIRILISR
CETDLLSIRAEFRKKFGKSLYSSLQDAVKGDCQSALLALCRAEDM
No structure; 345 residues

>sp|Q9UJ72|ANX10_HUMAN Annexin A10 OS=Homo sapiens OX=9606 GN=ANXA10 PE=1
SV=3
MFCGDYVQGTIFPAPNFNPIMDAQMLGGALQGFDCDKDMLINILTQRCNAQRMMIAEAYQ
SMYGRDLIGDMREQLSDHFKDVMAGLMYPPPLYDAHELWHAMKGVGTDENCLIEILASRT
NGEIFQMREAYCLQYSNNLQEDIYSETSGHFRDTLMNLVQGTREEGYTDPAMAAQDAMVL
WEACQQKTGEHKTMLQMILCNKSYQQLRLVFQEFQNISGQDMVDAINECYDGYFQELLVA
IVLCVRDKPAYFAYRLYSAIHDFGFHNKTVIRILIARSEIDLLTIRKRYKERYGKSLFHD
IRNFASGHYKKALLAICAGDAEDY
No structure; 324 residues

>sp|P50995|ANX11_HUMAN Annexin A11 OS=Homo sapiens OX=9606 GN=ANXA11 PE=1
SV=1
MSYPGYPPPPGGYPPAAPGGGPWGGAAYPPPPSMPPIGLDNVATYAGQFNQDYLSGMAAN
MSGTFGGANMPNLYPGAPGAGYPPVPPGGFGQPPSAQQPVPPYGMYPPPGGNPPSRMPSY
PPYPGAPVPGQPMPPPGQQPPGAYPGQPPVTYPGQPPVPLPGQQQPVPSYPGYPGSGTVT
PAVPPTQFGSRGTITDAPGFDPLRDAEVLRKAMKGFGTDEQAIIDCLGSRSNKQRQQILL
SFKTAYGKDLIKDLKSELSGNFEKTILALMKTPVLFDIYEIKEAIKGVGTDEACLIEILA
SRSNEHIRELNRAYKAEFKKTLEEAIRSDTSGHFQRLLISLSQGNRDESTNVDMSLAQRD
AQELYAAGENRLGTDESKFNAVLCSRSRAHLVAVFNEYQRMTGRDIEKSICREMSGDLEE
GMLAVVKCLKNTPAFFAERLNKAMRGAGTKDRTLIRIMVSRSETDLLDIRSEYKRMYGKS

LYHDISGDTSGDYRKILLKICGGND
No structure; 505 residues

>sp|P27216|ANX13_HUMAN Annexin A13 OS=Homo sapiens OX=9606 GN=ANXA13 PE=1 SV=3
MGNRHAKASSPQGFDVDRDAKKLNKACKGMGTNEAAIIEILSGRTSDERQQIKQKYKATY
GKELEEVLKSELSGNFEKTALALLDRPSEYAARQLQKAMKGLGTDESVLIEVLCTRTNKE
IIAIKEAYQRLFDRSLESDVKGDTSGNLKKILVSLLQANRNEGDDVDKDLAGQDAKDLYD
AGEGRWGTDELAFNEVLAKRSYKQLRATFQAYQILIGKDIEEAIEEETSGDLQKAYLTLV
RCAQDCEDYFAERLYKSMKGAGTDEETLIRIVVTRAEVDLQGIKAKFQEKYQKSLSDMVR
SDTSGDFRKLLVALLH
PDB ID: 6B3I 316 residues

Amino acid sequences of Human S100 proteins

>sp|P23297|S10A1_HUMAN Protein S100-A1 OS=Homo sapiens OX=9606 GN=S100A1 PE=1 SV=2
MGSELETAMETLINVFHAHSGKEGDKYKLSKKELKELLQTELSGFLDAQKDVDAVDKVMK
ELDENGDGEVDFQEYVVLVAALTVACNNFFWENS
PDB ID: 2LP3; 94 residues

>sp|P29034|S10A2_HUMAN Protein S100-A2 OS=Homo sapiens OX=9606 GN=S100A2 PE=1 SV=3
MMCSSLEQALAVLVTTFHKYSCQEGDKFKLSKGEMKELLHKELPSFVGEKVDEEGLKKLM
GSLDENSDQQVDFQEYAVFLALITVMCNDFFQGCPDRP
PDB ID: 4DUQ; 98 residues

>sp|P33764|S10A3_HUMAN Protein S100-A3 OS=Homo sapiens OX=9606 GN=S100A3 PE=1 SV=1
MARPLEQAVAAIVCTFQEYAGRCGDKYKLCQAELKELLQKELATWTPTEFRECDYNKFMS
VLDTNKDCEVDFVEYVRSLACLCLYCHEYFKDCPSEPPCSQ
PDB ID: 3NSK; 101 residues

>sp|P26447|S10A4_HUMAN Protein S100-A4 OS=Homo sapiens OX=9606 GN=S100A4 PE=1 SV=1
MACPLEKALDVMVSTFHKYSGKEGDKFKLNKSELKELLTRELPSFLGKRTDEAAFQKLMS
NLDSNRDNEVDFQEYCVFLSCIAMMCNEFFEGFPDKQPRKK
PDB ID: 2MRD; 101 residues

>sp|P33763|S10A5_HUMAN Protein S100-A5 OS=Homo sapiens OX=9606 GN=S100A5 PE=1 SV=2
METPLEKALTTMVTTFHKYSGREGSKLTLSRKELKELIKKELCLGEMKESSIDDLMKSLD
KNSDQEIDFKEYSVFLTMLCMAYNDFFLEDNK
PDB ID: 2KAX; 92 residues

>sp|P06703|S10A6_HUMAN Protein S100-A6 OS=Homo sapiens OX=9606 GN=S100A6 PE=1 SV=1
MACPLDQAIGLLVAIFHKYSGREGDKHTLSKKELKELIQKELTIGSKLQDAEIARLMEDL
DRNKDQEVNFQEYVTFLGALALIYNEALKG
PDB ID: 1K8U; 90 residues

>sp|P31151|S10A7_HUMAN Protein S100-A7 OS=Homo sapiens OX=9606 GN=S100A7 PE=1 SV=4
MSNTQAERSIIGMIDMFHKYTRRDDKIEKPSLLTMMKENFPNFLSACDKKGTNYLADVFE
KKDKNEDKKIDFSEFLSLLGDIATDYHKQSHGAAPCSGGSQ
PDB ID: 1PSR; 101 residues

>sp|Q86SG5|S1A7A_HUMAN Protein S100-A7A OS=Homo sapiens OX=9606 GN=S100A7A PE=1 SV=3
MSNTQAERSIIGMIDMFHKYTGRDGKIEKPSLLTMMKENFPNFLSACDKKGIHYLATVFE
KKDKNEDKKIDFSEFLSLLGDIAADYHKQSHGAAPCSGGSQ
PDB ID: 4AQI; 101 residues

>sp|Q5SY68|S1A7B_HUMAN Protein S100-A7-like 2 OS=Homo sapiens OX=9606 GN=S100A7L2 PE=1 SV=1
MNIPLGEKVMLDIVAMFRQYSGDDGRMDMPGLVNLMKENFPNFLSGCEKSDMDYLSNALE
KKDDNKDKKVNYSEFLSLLGDITIDHHKIMHGVAPCSGGSQ
No structure; 101 residues

>sp|P05109|S10A8_HUMAN Protein S100-A8 OS=Homo sapiens OX=9606 GN=S100A8 PE=1 SV=1
MLTELEKALNSIIDVYHKYSLIKGNFHAVYRDDLKKLLETECPQYIRKKGADVWFKELDI
NTDGAVNFQEFLILVIKMGVAAHKKSHEESHKE
PDB ID: 1MR8; 93 residues

>sp|P06702|S10A9_HUMAN Protein S100-A9 OS=Homo sapiens OX=9606 GN=S100A9 PE=1 SV=1
MTCKMSQLERNIETIINTFHQYSVKLGHPDTLNQGEFKELVRKDLQNFLKKENKNEKVIE
HIMEDLDTNADKQLSFEEFIMLMARLTWASHEKMHEGDEGPGHHHKPGLGEGTP
PDB ID: 5I8N; 114 residues

>sp|P60903|S10AA_HUMAN Protein S100-A10 OS=Homo sapiens OX=9606 GN=S100A10 PE=1 SV=2
MPSQMEHAMETMMFTFHKFAGDKGYLTKEDLRVLMEKEFPGFLENQKDPLAVDKIMKDLD
QCRDGKVGFQSFFSLIAGLTIACNDYFVVHMKQKGKK
PDB ID: 4FTG; 97 residues

>sp|P31949|S10AB_HUMAN Protein S100-A11 OS=Homo sapiens OX=9606 GN=S100A11 PE=1 SV=2
MAKISSPTETERCIESLIAVFQKYAGKDGYNYTLSKTEFLSFMNTELAAFTKNQKDPGVL
DRMMKKLDTNSDGQLDFSEFLNLIGGLAMACHDSFLKAVPSQKRT
PDB ID: 2LUC; 105 residues

>sp|P80511|S10AC_HUMAN Protein S100-A12 OS=Homo sapiens OX=9606 GN=S100A12 PE=1 SV=2
MTKLEEHLEGIVNIFHQYSVRKGHFDTLSKGELKQLLTKELANTIKNIKDKAVIDEIFQG
LDANQDEQVDFQEFISLVAIALKAAHYHTHKE
PDB ID: 2M9G; 92 residues

>sp|Q99584|S10AD_HUMAN Protein S100-A13 OS=Homo sapiens OX=9606 GN=S100A13 PE=1 SV=1
MAAEPLTELEESIETVVTTFFTFARQEGRKDSLSVNEFKELVTQQLPHLLKDVGSLDEKM
KSLDVNQDSELKFNEYWRLIGELAKEIRKKKDLKIRKK
PDB ID: 1YUS; 98 residues

>sp|Q9HCY8|S10AE_HUMAN Protein S100-A14 OS=Homo sapiens OX=9606 GN=S100A14 PE=1 SV=1
MGQCRSANAEDAQEFSDVERAIETLIKNFHQYSVEGGKETLTPSELRDLVTQQLPHLMPS
NCGLEEKIANLGSCNDSKLEFRSFWELIGEAAKSVKLERPVRGH
PDB ID: 2M0R; 104 residues

>sp|Q96FQ6|S10AG_HUMAN Protein S100-A16 OS=Homo sapiens OX=9606 GN=S100A16 PE=1 SV=1
MSDCYTELEKAVIVLVENFYKYVSKYSLVKNKISKSSFREMLQKELNHMLSDTGNRKAAD
KLIQNLDANHDGRISFDEYWTLIGGITGPIAKLIHEQEQQSSS
PDB ID: 2L50; 103 residues

>sp|P04271|S100B_HUMAN Protein S100-B OS=Homo sapiens OX=9606 GN=S100B PE=1 SV=2
MSELEKAMVALIDVFHQYSGREGDKHKLKKSELKELINNELSHFLEEIKEQEVVDKVMET
LDNDGDGECDFQEFMAFVAMVTTACHEFFEHE
PDB ID: 1UWO; 92 residues

>sp|P29377|S100G_HUMAN Protein S100-G OS=Homo sapiens OX=9606 GN=S100G PE=3 SV=2
MSTKKSPEELKRIFEKYAAKEGDPDQLSKDELKLLIQAEFPSLLKGPNTLDDLFQELDKN
GDGEVSFEEFQVLVKKISQ
No structure; 79 residues

>sp|P25815|S100P_HUMAN Protein S100-P OS=Homo sapiens OX=9606 GN=S100P PE=1 SV=2
MTELETAMGMIIDVFSRYSGSEGSTQTLTKGELKVLMEKELPGFLQSGKDKDAVDKLLKD
LDANGDAQVDFSEFIVFVAAITSACHKYFEKAGLK
PDB ID: 1OZO; 95 residues

>sp|Q8WXG8|S100Z_HUMAN Protein S100-Z OS=Homo sapiens OX=9606 GN=S100Z PE=1 SV=4
MPTQLEMAMDTMIRIFHRYSGKERKRFKLSKGELKLLLQRELTEFLSCQKETQLVDKIVQ
DLDANKDNEVDFNEFVVMVAALTVACNDYFVEQLKKKGK
PDB ID: 5HYD; 99 residues

>sp|Q5QJ38|TCHL1_HUMAN Trichohyalin-like protein 1 (Protein S100-A17) OS=Homo sapiens OX=9606 GN=TCHHL1 PE=2 SV=1
MPQLLRNVLCVIETFHKYASEDSNGATLTGRELKQLIQGEFGDFFQPCVLHAVEKNSNLL
NIDSNGIISFDEFVLAIFNLLNLCYLDIKSLLSSELRQVTKPEKEKLDDVDVQATTGDGQ
WTVGTSPTQEKRMLPSGMASSSQLIPEESGAVGNNRVDPWREAKTHNFPGEASEHNDPKN
KHLEGDEQSQEVAQDIQTTEDNEGQLKTNKPMAGSKKTSSPTERKGQDKEISQEGDEPAR
EQSVSKIRDQFGEQEGNLATQSSPPKEATQRPCEDQEVRTEKEKHSNIQEPPLQREDEPS
SQHADLPEQAAARSPSQTQKSTDSKDVCRMFDTQEPGKDADQTPAKTKNLGEPEDYGRTS
ETQEKECETKDLPVQYGSRNGSETSDMRDERKERRGPEAHGTAGQKERDRKTRPLVLETQ
TQDGKYQELQGLSKSKDAEKGSETQYLSSEGGDQTHPELEGTAVSGEEAEHTKEGTAEAF
VNSKNAPAAERTLGARERTQDLAPLEKQSVGENTRVTKTHDQPVEEEDGYQGEDPESPFT
QSDEGSSETPNSLASEEGNSSSETGELPVQGDSQSQGDQHGESVQGGHNNNPDTQRQGTP
GEKNRALEAVVPAVRGEDVQLTEDQEQPARGEHKNQGPGTKGPGAAVEPNGHPEAQESTA
GDENRKSLEIEITGALDEDFTDQLSLMQLPGKGDSRNELKVQGPSSKEEKGRATEAQNTL
LESLDEDNSASLKIQLETKEPVTSEEEDESPQELAGEGGDQKSPAKKEHNSSVPWSSLEK
QMQRDQEPCSVERGAVYSSPLYQYLQEKILQQTNVTQEEHQKQVQIAQASGPELCSVSLT
SEISDCSVFFNYSQASQPYTRGLPLDESPAGAQETPAPQALEDKQGHPQRERLVLQREAS
TTKQ
No structure; 904 residues

>sp|Q86YZ3|HORN_HUMAN Hornerin (Protein S100-A18) OS=Homo sapiens OX=9606 GN=HRNR PE=1 SV=2
MPKLLQGVITVIDVFYQYATQHGEYDTLNKAELKELLENEFHQILKNPNDPDTVDIILQS
LDRDHNKKVDFTEYLLMIFKLVQARNKIIGKDYCQVSGSKLRDDTHQHQEEQEETEKEEN
KRQESSFSHSSWSAGENDSYSRNVRGSLKPGTESISRRLSFQRDFSGQHNSYSGQSSSYG
EQNSDSHQSSGRGQCGSGSGQSPNYGQHGSGSGQSSSNDTHGSGSGQSSGFSQHKSSSGQ
SSGYSQHGSGSGHSSGYGQHGSRSGQSSRGERHRSSSGSSSSYGQHGSGSRQSLGHGRQG
SGSRQSPSHVRHGSGSGHSSSHGQHGSGSSYSYSRGHYESGSGQTSGFGQHESGSGQSSG
YSKHGSGSGHSSSQGQHGSTSGQASSSGQHGSSSRQSSSYGQHESASRHSSGRGQHSSGS
GQSPGHGQRGSGSGQSPSSGQHGTGFGRSSSSGPYVSGSGYSSGFGHHESSSEHSSGYTQ
HGSGSGHSSGHGQHGSRSGQSSRGERQGSSAGSSSSYGQHGSGSRQSLGHSRHGSGSGQS

PSPSRGRHESGSRQSSSYGPHGYGSGRSSSRGPYESGSGHSSGLGHQESRSGQSSGYGQH
GSSSGHSSTHGQHGSTSGQSSSCGQHGATSGQSSSHGQHGSGSSQSSRYGQQGSGSGQSP
SRGRHGSDFGHSSSYGQHGSGSGWSSSNGPHGSVSGQSSGFGHKSGSGQSSGYSQHGSGS
SHSSGYRKHGSRSGQSSRSEQHGSSSGLSSSYGQHGSGSHQSSGHGRQGSGSGHSPSRVR
HGSSSGHSSSHGQHGSGTSCSSSCGHYESGSGQASGFGQHESGSGQGYSQHGSASGHFSS
QGRHGSTSGQSSSSGQHDSSSGQSSSYGQHESASHHASGRGRHGSGSGQSPGHGQRGSGS
GQSPSYGRHGSGSGRSSSSGRHGSGSGQSSGFGHKSSSGQSSGYTQHGSGSGHSSSYEQH
GSRSGQSSRSEQHGSSSGSSSSYGQHGSGSRQSLGHGQHGSGSGQSPSPSRGRHGSGSGQ
SSSYGPYRSGSGWSSSRGPYESGSGHSSGLGHRESRSGQSSGYGQHGSSSGHSSTHGQHG
STSGQSSSCGQHGASSGQSSSHGQHGSGSSQSSGYGRQGSGSGQSPGHGQRGSGSRQSPS
YGRHGSGSGRSSSSGQHGSGLGESSGFGHHESSSGQSSSYSQHGSGSGHSSGYGQHGSRS
GQSSRGERHGSSSGSSSHYGQHGSGSRQSSGHGRQGSGSGHSPSRGRHGSGLGHSSSHGQ
HGSGSGRSSSRGPYESRSGHSSVFGQHESGSGHSSAYSQHGSGSGHFCSQGQHGSTSGQS
STFDQEGSSTGQSSSYGHRGSGSSQSSGYGRHGAGSGQSPSRGRHGSGSGHSSSYGQHGS
GSGWSSSSGRHGSGSGQSSGFGHHESSSWQSSGCTQHGSGSGHSSSYEQHGSRSGQSSRG
ERHGSSSGSSSSYGQHGSGSRQSLGHGQHGSGSGQSPSPSRGRHGSGSGQSSSYSPYGSG
SGWSSSRGPYESGSSHSSGLGHRESRSGQSSGYGQHGSSSGHSSTHGQHGSTSGQSSSCG
QHGASSGQSSSHGQHGSGSSQSSGYGRQGSGSGQSPGHGQRGSGSRQSPSYGRHGSGSGR
SSSSGQHGSGLGESSGFGHHESSSGQSSSYSQHGSGSGHSSGYGQHGSRSGQSSRGERHG
SSSRSSSRYGQHGSGSRQSSGHGRQGSGSGQSPSRGRHGSGLGHSSSHGQHGSGSGRSSS
RGPYESRSGHSSVFGQHESGSGHSSAYSQHGSGSGHFCSQGQHGSTSGQSSTFDQEGSST
GQSSSHGQHGSGSSQSSSYGQQGSGSGQSPSRGRHGSGSGHSSSYGQHGSGSGWSSSSGR
HGSGSGQSSGFGHHESSSWQSSGYTQHGSGSGHSSSYEQHGSRSGQSSRGEQHGSSSGSS
SSYGQHGSGSRQSLGHGQHGSGSGQSPSPSRGRHGSGSGQSSSYGPYGSGSGWSSSRGPY
ESGSGHSSGLGHRESRSGQSSGYGQHGSSSGHSSTHGQHGSASGQSSSCGQHGASSGQSS
SHGQHGSGSSQSSGYGRQGSGSGQSPGHGQRGSGSRQSPSYGRHGSGSGRSSSSGQHGPG
LGESSGFGHHESSSGQSSSYSQHGSGSGHSSGYGQHGSRSGQSSRGERHGSSSGSSSRYG
QHGSGSRQSSGHGRQGSGSGHSPSRGRHGSGSGHSSSHGQHGSGSGRSSSRGPYESRSGH
SSVFGQHESGSGHSSAYSQHGSGSGHFCSQGQHGSTSGQSSTFDQEGSSTGQSSSHGQHG
SGSSQSSSYGQQGSGSGQSPSRGRHGSGSGHSSSYGQHGSGSGWSSSSGRHGSGSGQSSG
FGHHESSSWQSSGYTQHGSGSGHSSSYEQHGSRSGQSSRGERHGSSSGSSSSYGQHGSGS
RQSLGHGQHGSGSGQSPSPSRGRHGSGSGQSSSYSPYGSGSGWSSSRGPYESGSGHSSGL
GHRESRSGQSSGYGQHGSSSGHSSTHGQHGSTSGQSSSCGQHGASSGQSSSHGQHGSGSS
QSSGYGRQGSGSGQSPGHGQRGSGSRQSPSYGRHGSGSGRSSSSGQHGSGLGESSGFGHH
ESSSGQSSSYSQHGSGSGHSSGYGQHGSRSGQSSRGERHGSSSGSSSHYGQHGSGSRQSS
GHGRQGSGSGQSPSRGRHGSGLGHSSSHGQHGSGSGRSSSRGPYESRLGHSSVFGQHESG
SGHSSAYSQHGSGSGHFCSQGQHGSTSGQSSTFDQEGSSTGQSSSYGHRGSGSSQSSGYG
RHGAGSGQSLSHGRHGSGSGQSSSYGQHGSGSGQSSGYSQHGSGSGQDGYSYCKGGSNHD
GGSSGSYFLSFPSSTSPYEYVQEQRCYFYQ

No structure; 2850 residues

UniProt IDs of human annexins and S100 proteins

Annexins

P04083
P07355
P12429
P09525
P08758
P08133
P20073
P13928
O76027
Q9UJ72
P50995
P27216

S100 proteins

P23297
P29034
P33764
P26447
P33763
P06703
P31151
Q86SG5
Q5SY68
P05109
P06702
P60903
P31949
P80511
Q99584
Q9HCY8
Q96FQ6
P04271
P29377
P25815
Q8WXG8
Q5QJ38
Q86YZ3

Multiple sequence alignment of human annexins

CLUSTAL O(1.2.4) multiple sequence alignment

```
sp|P04083|ANXA1_HUMAN      ------------------------------------------------------------      0
sp|P07355|ANXA2_HUMAN      ------------------------------------------------------------      0
sp|P12429|ANXA3_HUMAN      ------------------------------------------------------------      0
sp|P09525|ANXA4_HUMAN      ------------------------------------------------------------      0
sp|P08758|ANXA5_HUMAN      ------------------------------------------------------------      0
sp|P08133|ANXA6_HUMAN      ------------------------------------------------------------      0
sp|P20073|ANXA7_HUMAN      MSYPGYPPTGYPPFPGYPPAGQESSFPPSGQYPYPSGFPPMGGGAYPQV-------PSSG      53
sp|P13928|ANXA8_HUMAN      ------------------------------------------------------------      0
sp|O76027|ANXA9_HUMAN      ------------------------------------------------------------      0
sp|Q9UJ72|ANX10_HUMAN      ------------------------------------------------------------      0
sp|P50995|ANX11_HUMAN      MSYPGYPPP----PGGYPPAAPGGGPWGGAAYPPPPSMPPIGLDNVATYAGQFNQDYLSG      56
sp|P27216|ANX13_HUMAN      ------------------------------------------------------------      0

sp|P04083|ANXA1_HUMAN      ------------------------------------------------------------      0
sp|P07355|ANXA2_HUMAN      ------------------------------------------------------------      0
sp|P12429|ANXA3_HUMAN      ------------------------------------------------------------      0
sp|P09525|ANXA4_HUMAN      ------------------------------------------------------------      0
sp|P08758|ANXA5_HUMAN      ------------------------------------------------------------      0
sp|P08133|ANXA6_HUMAN      ------------------------------------------------------------      0
sp|P20073|ANXA7_HUMAN      YPGAGGYPAPGGYPAPGGYPGAPQPGGAPSYPGVPPGQGFGVP---------------PG      98
sp|P13928|ANXA8_HUMAN      ------------------------------------------------------------      0
sp|O76027|ANXA9_HUMAN      ------------------------------------------------------------      0
sp|Q9UJ72|ANX10_HUMAN      ------------------------------------------------------------      0
sp|P50995|ANX11_HUMAN      MAA-NMSGTFGGANMPNLYPGAPGAG----YPPVPPG-GFGQPPSAQQPVPPYGMYPPPG      110
sp|P27216|ANX13_HUMAN      ------------------------------------------------------------      0

sp|P04083|ANXA1_HUMAN      -------------------------------------------------MA-MVSEFLKQ      10
sp|P07355|ANXA2_HUMAN      -------------------------------------------------MS-TVHEILCK      10
sp|P12429|ANXA3_HUMAN      ------------------------------------------------------------      0
sp|P09525|ANXA4_HUMAN      ------------------------------------------------------------      0
sp|P08758|ANXA5_HUMAN      ------------------------------------------------------------      0
sp|P08133|ANXA6_HUMAN      ------------------------------------------------------------      0
sp|P20073|ANXA7_HUMAN      GAGFSG---YPQPPSQSYGGGPAQVPLPGGFPG----GQMPSQYPGGQPTY--P-SQINT      148
sp|P13928|ANXA8_HUMAN      -----------------------------------------------------------M      1
sp|O76027|ANXA9_HUMAN      -----------------------------------------MSVTGGKMAPSLTQEILSH      19
sp|Q9UJ72|ANX10_HUMAN      ------------------------------------------------------------      0
sp|P50995|ANX11_HUMAN      GNPPSRMPSYPPYPGAPVPGQ--PMPPPGQQPPGAYPGQPPVTYPGQPPVP-LPGQQQPV      167
sp|P27216|ANX13_HUMAN      ------------------------------------------------------------      0

sp|P04083|ANXA1_HUMAN      AWFIENEE----Q-EYVQTVKSSKGGPGSAVSPYPTFNPSSDVAALHKAIMVKGVDEATI      65
sp|P07355|ANXA2_HUMAN      LSL--------------EGDHSTPPSAYGSVKAYTNFDAERDALNIETAIKTKGVDEVTI      56
sp|P12429|ANXA3_HUMAN      -------------------MASIWVGHRGTVRDYPDFSPSVDAEAIQKAIRGIGTDEKML      41
sp|P09525|ANXA4_HUMAN      -----------------------MATKGGTVKAASGFNAMEDAQTLRKAMKGLGTDEDAI      37
sp|P08758|ANXA5_HUMAN      ----------------------MAQVLRGTVTDFPGFDERADAETLRKAMKGLGTDEESI      38
sp|P08133|ANXA6_HUMAN      -----------------MAKPAQGAKYRGSIHDFPGFDPNQDAEALYTAMKGFGSDKEAI      43
sp|P20073|ANXA7_HUMAN      DSFSSYPVFSPVSLDYSSEPATVTQVTQGTIRPAANFDAIRDAEILRKAMKGFGTDEQAI      208
sp|P13928|ANXA8_HUMAN      AW-----------------WKSWIEQEGVTVKSSSHFNPDPDAETLYKAMKGIGTNEQAI      44
sp|O76027|ANXA9_HUMAN      ---------------LGLASKTAAWGTLGTLRTFLNFSVDKDAQRLLRAITGQGVDRSAI      64
sp|Q9UJ72|ANX10_HUMAN      --------------------MFCGDYVQGTIFPAPNFNPIMDAQMLGGALQGFDCDKDML      40
sp|P50995|ANX11_HUMAN      PSYPGYPG----SGTVTPAVPPTQFGSRGTITDAPGFDPLRDAEVLRKAMKGFGTDEQAI      223
sp|P27216|ANX13_HUMAN      -----------------------MGNRHAKASSPQGFDVDRDAKKLNKACKGMGTNEAAI      37
                                                               *.   *.  :  *    . :.  :

sp|P04083|ANXA1_HUMAN      IDILTKRNNAQRQQIKAAYLQETGKPLDETLKKALTGHLEEVVLALLKTPAQFDADELRA      125
sp|P07355|ANXA2_HUMAN      VNILTNRSNAQRQDIAFAYQRRTKKELASALKSALSGHLETVILGLLKTPAQYDASELKA      116
sp|P12429|ANXA3_HUMAN      ISILTERSNAQRQLIVKEYQAAYGKELKDDLKGDLSGHFEHLMVALVTPPAVFDAKQLKK      101
sp|P09525|ANXA4_HUMAN      ISVLAYRNTAQRQEIRTAYKSTIGRDLIDDLKSELSGNFEQVIVGMMTPTVLYDVQELRR      97
sp|P08758|ANXA5_HUMAN      LTLLTSRSNAQRQEISAAFKTLFGRDLLDDLKSELTGKFEKLIVALMKPSRLYDAYELKH      98
sp|P08133|ANXA6_HUMAN      LDIITSRSNRQRQEVCQSYKSLYGKDLIADLKYELTGKFERLIVGLMRPPAYCDAKEIKD      103
sp|P20073|ANXA7_HUMAN      VDVVANRSNDQRQKIKAAFKTSYGKDLIKDLKSELSGNMEELILALFMPPTYYDAWSLRK      268
sp|P13928|ANXA8_HUMAN      IDVLTKRSNTQRQQIAKSFKAQFGKDLTETLKSELSGKFERLIVALMYPPYRYEAKELHD      104
sp|O76027|ANXA9_HUMAN      VDVLTNRSREQRQLISRNFQERTQQDLMKSLQAALSGNLERIVMALLQPTAQFDAQELRT      124
sp|Q9UJ72|ANX10_HUMAN      INILTQRCNAQRMMIAEAYQSMYGRDLIGDMREQLSDHFKDVMAGLMYPPPLYDAHELWH      100
sp|P50995|ANX11_HUMAN      IDCLGSRSNKQRQQILLSFKTAYGKDLIKDLKSELSGNFEKTILALMKTPVLFDIYEIKE      283
sp|P27216|ANX13_HUMAN      IEILSGRTSDERQQIKQKYKATYGKELEEVLKSELSGNFEKTALALLDRPSEYAARQLQK      97
                           :  :  *   :*  :   :     : *    ::   *:.:::  .:.         .:

sp|P04083|ANXA1_HUMAN      AMKGLGTDEDTLIEILASRTNKEIRDINRVYREELKRDLAKDITSDTSGDFRNALLSLAK      185
sp|P07355|ANXA2_HUMAN      SMKGLGTDEDSLIEIICSRTNQELQEINRVYKEMYKTDLEKDIISDTSGDFRKLMVALAK      176
sp|P12429|ANXA3_HUMAN      SMKGAGTNEDALIEILTTRTSRQMKDISQAYYTVYKKSLGDDISSETSGDFRKALLTLAD      161
sp|P09525|ANXA4_HUMAN      AMKGAGTDEGCLIEILASRTPEEIRRISQTYQQQYGRSLEDDIRSDTSFMFQRVLVSLSA      157
sp|P08758|ANXA5_HUMAN      ALKGAGTNEKVLTEIIASRTPEELRAIKQVYEEEYGSSLEDDVVGDTSGYYQRMLVVLLQ      158
sp|P08133|ANXA6_HUMAN      AISGIGTDEKCLIEILASRTNEQMHQLVAAYKDAYERDLEADIIGDTSGHFQKMLVVLLQ      163
```

```
sp|P20073|ANXA7_HUMAN      AMQGAGTQERVLIEILCTRTNQEIREIVRCYQSEFGRDLEKDIRSDTSGHFERLLVSMCQ        328
sp|P13928|ANXA8_HUMAN      AMKGLGTKEGVIIEILASRTKNQLREIMKAYEEDYGSSLEEDIQADTSGYLERILVCLLQ        164
sp|O76027|ANXA9_HUMAN      ALKASDSAVDVAIEILATRTPPQLQECLAVYKHNFQVEAVDDITSETSGILQDLLLALAK        184
sp|Q9UJ72|ANX10_HUMAN      AMKGVGTDENCLIEILASRTNGEIFQMREAYCLQYSNNLQEDIYSETSGHFRDTLMNLVQ        160
sp|P50995|ANX11_HUMAN      AIKGVGTDEACLIEILASRSNEHIRELNRAYKAEFKKTLEEAIRSDTSGHFQRLLISLSQ        343
sp|P27216|ANX13_HUMAN      AMKGLGTDESVLIEVLCTRTNKEIIAIKEAYQRLFDRSLESDVKGDTSGNLKKILVSLLQ        157
                            ::.. .:      *:: :*:  .:      *           : .:**   .  :: :

sp|P04083|ANXA1_HUMAN      GDRSEDFGV-NEDLADSDARALYEAGERRKGTDVNVFNTILTTRSYPQLRRVFQKYTKYS        244
sp|P07355|ANXA2_HUMAN      GRRAEDGSVIDYELIDQDARDLYDAGVKRKGTDVPKWISIMTERSVPHLQKVFDRYKSYS        236
sp|P12429|ANXA3_HUMAN      GRRDE-SLKVDEHLAKQDAQILYKAGENRWGTDEDKFTEILCLRSFPQLKLTFDEYRNIS        220
sp|P09525|ANXA4_HUMAN      GGRDE-GNYLDDALVRQDAQDLYEAGEKKWGTDEVKFLTVLCSRNRNHLLHVFDEYKRIS        216
sp|P08758|ANXA5_HUMAN      ANRDP-DAGIDEAQVEQDAQALFQAGELKWGTDEEKFITIFGTRSVSHLRKVFDKYMTIS        217
sp|P08133|ANXA6_HUMAN      GTREE-DDVVSEDLVQQDVQDLYEAGELKWGTDEAQFIYILGNRSKQHLRLVFDEYLKTT        222
sp|P20073|ANXA7_HUMAN      GNRDE-NQSINHQMAQEDAQRLYQAGEGRLGTDESCFNMILATRSFPQLRATMEAYSRMA        387
sp|P13928|ANXA8_HUMAN      GSRDDVSSFVDPGLALQDAQDLYAAGEKIRGTDEMKFITILCTRSATHLLRVFEEYEKIA        224
sp|O76027|ANXA9_HUMAN      GGRDSYSGIIDYNLAEQDVQALQRAEG---PSREETWVPVFTQRNPEHLIRVFDQYQRST        241
sp|Q9UJ72|ANX10_HUMAN      GTREE--GYTDPAMAAQDAMVLWEACQQKTGEHKTMLQMILCNKSYQQLRLVFQEFQNIS        218
sp|P50995|ANX11_HUMAN      GNRDE-STNVDMSLAQRDAQELYAAGENRLGTDESKFNAVLCSRSRAHLVAVFNEYQRMT        402
sp|P27216|ANX13_HUMAN      ANRNE-GDDVDKDLAGQDAKDLYDAGEGRWGTDELAFNEVLAKRSYKQLRATFQAYQILI        216
                            . *       .      *.  *  *              ::  :.  :*  .:: :

sp|P04083|ANXA1_HUMAN      KHDMNKVLDLELKGDIEKCLTAIVKCATSKPAFFAEKLHQAMKGVGTRHKALIRIMVSRS        304
sp|P07355|ANXA2_HUMAN      PYDMLESIRKEVKGDLENAFLNLVQCIQNKPLYFADRLYDSMKGKGTRDKVLIRIMVSRS        296
sp|P12429|ANXA3_HUMAN      QKDIVDSIKGELSGHFEDLLLAIVNCVRNTPAFLAERLHRALKGIGTDEFTLNRIMVSRS        280
sp|P09525|ANXA4_HUMAN      QKDIEQSIKSETSGSFEDALLAIVKCMRNKSAYFAEKLYKSMKGLGTDDNTLIRVMVSRA        276
sp|P08758|ANXA5_HUMAN      GFQIEETIDRETSGNLEQLLLAVVKSIRSIPAYLAETLYYAMKGAGTDDHTLIRVMVSRS        277
sp|P08133|ANXA6_HUMAN      GKPIEASIRGELSGDFEKLMLAVVKCIRSTPEYFAERLFKAMKGLGTRDNTLIRIMVSRS        282
sp|P20073|ANXA7_HUMAN      NRDLLSSVSREFSGYVESGLKTILQCALNRPAFFAERLYYAMKGAGTDDSTLVRIVVTRS        447
sp|P13928|ANXA8_HUMAN      NKSIEDSIKSETHGSLEEAMLTVVKCTQNLHSYFAERLYYAMKGAGTRDGTLIRNIVSRS        284
sp|O76027|ANXA9_HUMAN      GQELEEAVQNRFHGDAQVALLGLASVIKNTPLYFADKLHQALQETEPNYQVLIRILISRC        301
sp|Q9UJ72|ANX10_HUMAN      GQDMVDAINECYDGYFQELLVAIVLCVRDKPAYFAYRLYSAIHDFGFHNKTVIRILIARS        278
sp|P50995|ANX11_HUMAN      GRDIEKSICREMSGDLEEGMLAVVKCLKNTPAFFAERLNKAMRGAGTKDRTLIRIMVSRS        462
sp|P27216|ANX13_HUMAN      GKDIEEAIEEETSGDLQKAYLTLVRCAQDCEDYFAERLYKSMKGAGTDEETLIRIVVTRA        276
                              :   :     *  :     :     .   ::*  *  :::       .: * :::*.

sp|P04083|ANXA1_HUMAN      EIDMNDIKAFYQKMYGISLCQAILDETKGDYEKILVALCGGN------------------        346
sp|P07355|ANXA2_HUMAN      EVDMLKIRSEFKRKYGKSLYYYIQQDTKGDYQKALLYLCGGDD-----------------        339
sp|P12429|ANXA3_HUMAN      EIDLLDIRTEFKKHYGYSLYSAIKSDTSGDYEITLLKICGGDD-----------------        323
sp|P09525|ANXA4_HUMAN      EIDMLDIRAHFKRLYGKSLYSFIKGDTSGDYRKVLLVLCGGDD-----------------        319
sp|P08758|ANXA5_HUMAN      EIDLFNIRKEFRKNFATSLYSMIKGDTSGDYKKALLLLCGEDD-----------------        320
sp|P08133|ANXA6_HUMAN      ELDMLDIREIFRTKYEKSLYSMIKNDTSGEYKKTLLKLSGGDDDAAGQFFPEAAQVAYQM        342
sp|P20073|ANXA7_HUMAN      EIDLVQIKQMFAQMYQKTLGTMIAGDTSGDYRRLLLAIVGQ-------------------        488
sp|P13928|ANXA8_HUMAN      EIDLNLIKCHFKKMYGKTLSSMIMEDTSGDYKNALLSLVGSDP-----------------        327
sp|O76027|ANXA9_HUMAN      ETDLLSIRAEFRKKFGKSLYSSLQDAVKGDCQSALLALCRAEDM----------------        345
sp|Q9UJ72|ANX10_HUMAN      EIDLLTIRKRYKERYGKSLFHDIRNFASGHYKKALLAICAGDAEDY--------------        324
sp|P50995|ANX11_HUMAN      ETDLLDIRSEYKRMYGKSLYHDISGDTSGDYRKILLKICGGND-----------------        505
sp|P27216|ANX13_HUMAN      EVDLQGIKAKFQEKYQKSLSDMVRSDTSGDFRKLLVALLH--------------------        316
                           * *:  *:  :   :  :*   :   ..*. .  *: :

sp|P04083|ANXA1_HUMAN      ------------------------------------------------------------        346
sp|P07355|ANXA2_HUMAN      ------------------------------------------------------------        339
sp|P12429|ANXA3_HUMAN      ------------------------------------------------------------        323
sp|P09525|ANXA4_HUMAN      ------------------------------------------------------------        319
sp|P08758|ANXA5_HUMAN      ------------------------------------------------------------        320
sp|P08133|ANXA6_HUMAN      WELSAVARVELKGTVRPANDFNPDADAKALRKAMKGLGTDEDTIIDIITHRSNVQRQQIR        402
sp|P20073|ANXA7_HUMAN      ------------------------------------------------------------        488
sp|P13928|ANXA8_HUMAN      ------------------------------------------------------------        327
sp|O76027|ANXA9_HUMAN      ------------------------------------------------------------        345
sp|Q9UJ72|ANX10_HUMAN      ------------------------------------------------------------        324
sp|P50995|ANX11_HUMAN      ------------------------------------------------------------        505
sp|P27216|ANX13_HUMAN      ------------------------------------------------------------        316


sp|P04083|ANXA1_HUMAN      ------------------------------------------------------------        346
sp|P07355|ANXA2_HUMAN      ------------------------------------------------------------        339
sp|P12429|ANXA3_HUMAN      ------------------------------------------------------------        323
sp|P09525|ANXA4_HUMAN      ------------------------------------------------------------        319
sp|P08758|ANXA5_HUMAN      ------------------------------------------------------------        320
sp|P08133|ANXA6_HUMAN      QTFKSHFGRDLMTDLKSEISGDLARLILGLMMPPAHYDAKQLKKAMEGAGTDEKALIEIL        462
sp|P20073|ANXA7_HUMAN      ------------------------------------------------------------        488
sp|P13928|ANXA8_HUMAN      ------------------------------------------------------------        327
sp|O76027|ANXA9_HUMAN      ------------------------------------------------------------        345
sp|Q9UJ72|ANX10_HUMAN      ------------------------------------------------------------        324
sp|P50995|ANX11_HUMAN      ------------------------------------------------------------        505
sp|P27216|ANX13_HUMAN      ------------------------------------------------------------        316


sp|P04083|ANXA1_HUMAN      ------------------------------------------------------------        346
sp|P07355|ANXA2_HUMAN      ------------------------------------------------------------        339
sp|P12429|ANXA3_HUMAN      ------------------------------------------------------------        323
```

```
sp|P09525|ANXA4_HUMAN      ------------------------------------------------------------      319
sp|P08758|ANXA5_HUMAN      ------------------------------------------------------------      320
sp|P08133|ANXA6_HUMAN      ATRTNAEIRAINEAYKEDYHKSLEDALSSDTSGHFRRILISLATGHREEGGENLDQARED      522
sp|P20073|ANXA7_HUMAN      ------------------------------------------------------------      488
sp|P13928|ANXA8_HUMAN      ------------------------------------------------------------      327
sp|O76027|ANXA9_HUMAN      ------------------------------------------------------------      345
sp|Q9UJ72|ANX10_HUMAN      ------------------------------------------------------------      324
sp|P50995|ANX11_HUMAN      ------------------------------------------------------------      505
sp|P27216|ANX13_HUMAN      ------------------------------------------------------------      316


sp|P04083|ANXA1_HUMAN      ------------------------------------------------------------      346
sp|P07355|ANXA2_HUMAN      ------------------------------------------------------------      339
sp|P12429|ANXA3_HUMAN      ------------------------------------------------------------      323
sp|P09525|ANXA4_HUMAN      ------------------------------------------------------------      319
sp|P08758|ANXA5_HUMAN      ------------------------------------------------------------      320
sp|P08133|ANXA6_HUMAN      AQVAAEILEIADTPSGDKTSLETRFMTILCTRSYPHLRRVFQEFIKMTNYDVEHTIKKEM      582
sp|P20073|ANXA7_HUMAN      ------------------------------------------------------------      488
sp|P13928|ANXA8_HUMAN      ------------------------------------------------------------      327
sp|O76027|ANXA9_HUMAN      ------------------------------------------------------------      345
sp|Q9UJ72|ANX10_HUMAN      ------------------------------------------------------------      324
sp|P50995|ANX11_HUMAN      ------------------------------------------------------------      505
sp|P27216|ANX13_HUMAN      ------------------------------------------------------------      316


sp|P04083|ANXA1_HUMAN      ------------------------------------------------------------      346
sp|P07355|ANXA2_HUMAN      ------------------------------------------------------------      339
sp|P12429|ANXA3_HUMAN      ------------------------------------------------------------      323
sp|P09525|ANXA4_HUMAN      ------------------------------------------------------------      319
sp|P08758|ANXA5_HUMAN      ------------------------------------------------------------      320
sp|P08133|ANXA6_HUMAN      SGDVRDAFVAIVQSVKNKPLFFADKLYKSMKGAGTDEKTLTRIMVSRSEIDLLNIRREFI      642
sp|P20073|ANXA7_HUMAN      ------------------------------------------------------------      488
sp|P13928|ANXA8_HUMAN      ------------------------------------------------------------      327
sp|O76027|ANXA9_HUMAN      ------------------------------------------------------------      345
sp|Q9UJ72|ANX10_HUMAN      ------------------------------------------------------------      324
sp|P50995|ANX11_HUMAN      ------------------------------------------------------------      505
sp|P27216|ANX13_HUMAN      ------------------------------------------------------------      316


sp|P04083|ANXA1_HUMAN      -------------------------------      346
sp|P07355|ANXA2_HUMAN      -------------------------------      339
sp|P12429|ANXA3_HUMAN      -------------------------------      323
sp|P09525|ANXA4_HUMAN      -------------------------------      319
sp|P08758|ANXA5_HUMAN      -------------------------------      320
sp|P08133|ANXA6_HUMAN      EKYDKSLHQAIEGDTSGDFLKALLALCGGED      673
sp|P20073|ANXA7_HUMAN      -------------------------------      488
sp|P13928|ANXA8_HUMAN      -------------------------------      327
sp|O76027|ANXA9_HUMAN      -------------------------------      345
sp|Q9UJ72|ANX10_HUMAN      -------------------------------      324
sp|P50995|ANX11_HUMAN      -------------------------------      505
sp|P27216|ANX13_HUMAN      -------------------------------      316
```

Percent Identity Matrix - created by Clustal2.1
#
#

1: sp|P04083|ANXA1_HUMAN	100.00	52.52	48.91	45.43	42.45	43.34	42.40	44.00	34.23	39.18	44.06	43.17
2: sp|P07355|ANXA2_HUMAN	52.52	100.00	47.37	48.90	45.31	46.46	41.32	44.65	39.64	37.38	44.97	42.09
3: sp|P12429|ANXA3_HUMAN	48.91	47.37	100.00	52.66	49.38	50.77	45.17	48.61	35.62	42.99	51.39	42.09
4: sp|P09525|ANXA4_HUMAN	45.43	48.90	52.66	100.00	57.68	53.92	48.90	55.49	35.76	43.71	56.74	46.52
5: sp|P08758|ANXA5_HUMAN	42.45	45.31	49.38	57.68	100.00	56.88	48.11	55.00	36.91	37.30	52.81	43.04
6: sp|P08133|ANXA6_HUMAN	43.34	46.46	50.77	53.92	56.88	100.00	43.96	51.08	33.44	41.98	56.62	43.99
7: sp|P20073|ANXA7_HUMAN	42.40	41.32	45.17	48.90	48.11	43.96	100.00	49.07	31.36	36.99	51.91	49.05
8: sp|P13928|ANXA8_HUMAN	44.00	44.65	48.61	55.49	55.00	51.08	49.07	100.00	34.47	37.07	51.84	48.10
9: sp|O76027|ANXA9_HUMAN	34.23	39.64	35.62	35.76	36.91	33.44	31.36	34.47	100.00	29.78	35.38	30.35
10: sp|Q9UJ72|ANX10_HUMAN	39.18	37.38	42.99	43.71	37.30	41.98	36.99	37.07	29.78	100.00	41.74	34.29
11: sp|P50995|ANX11_HUMAN	44.06	44.97	51.39	56.74	52.81	56.62	51.91	51.84	35.38	41.74	100.00	46.84
12: sp|P27216|ANX13_HUMAN	43.17	42.09	42.09	46.52	43.04	43.99	49.05	48.10	30.35	34.29	46.84	100.00

Multiple sequence alignment of human S100 proteins

```
CLUSTAL O(1.2.4) multiple sequence alignment


sp|P23297|S10A1_HUMAN      -------------MGSELETAMETLINVFHAHSGKEG-DKYKLSKKELKELLQTELSGFL        46
sp|P29034|S10A2_HUMAN      ------------MMCSSLEQALAVLVTTFHKYSCQEG-DKFKLSKGEMKELLHKELPSFV        47
sp|P33764|S10A3_HUMAN      -------------MARPLEQAVAAIVCTFQEYAGRCG-DKYKLCQAELKELLQKELATWT        46
sp|P26447|S10A4_HUMAN      -------------MACPLEKALDVMVSTFHKYSGKEG-DKFKLNKSELKELLTRELPSFL        46
sp|P33763|S10A5_HUMAN      -------------METPLEKALTTMVTTFHKYSGREG-SKLTLSRKELKELIKKELC--L        44
sp|P06703|S10A6_HUMAN      -------------MACPLDQAIGLLVAIFHKYSGREG-DKHTLSKKELKELIQKELT--I        44
sp|P31151|S10A7_HUMAN      ------------MSNTQAERSIIGMIDMFHKYTRRDD----KIEKPSLLTMMKENFPNFL        44
sp|Q86SG5|S1A7A_HUMAN      ------------MSNTQAERSIIGMIDMFHKYTGRDG----KIEKPSLLTMMKENFPNFL        44
sp|Q5SY68|S1A7B_HUMAN      ------------MNIPLGEKVMLDIVAMFRQYSGDDG----RMDMPGLVNLMKENFPNFL        44
sp|P05109|S10A8_HUMAN      -------------MLTELEKALNSIIDVYHKYSLIKG-NFHAVYRDDLKKLLETECPQYI        46
sp|P06702|S10A9_HUMAN      ----------MTCKMSQLERNIETIINTFHQYSVKLG-HPDTLNQGEFKELVRKDLQNFL        49
sp|P60903|S10AA_HUMAN      -------------MPSQMEHAMETMMFTFHKFAGDKG----YLTKEDLRVLMEKEFPGFL        43
sp|P31949|S10AB_HUMAN      --------MAKISSPTETERCIESLIAVFQKYAGKDG-YNYTLSKTEFLSFMNTELAAFT        51
sp|P80511|S10AC_HUMAN      --------------MTKLEEHLEGIVNIFHQYSVRKG-HFDTLSKGELKQLLTKELANTI        45
sp|Q99584|S10AD_HUMAN      ---------MAAEPLTELEESIETVVTTFFTFARQEG-RKDSLSVNEFKELVTQQLPHLL        50
sp|Q9HCY8|S10AE_HUMAN      MGQCRSANAEDAQEFSDVERAIETLIKNFHQYSVE-G-GKETLTPSELRDLVTQQLPHLM        58
sp|Q96FQ6|S10AG_HUMAN      ----------MSDCYTELEKAVIVLVENFYKYVSKYSLVKNKISKSSFREMLQKELNHML        50
sp|P04271|S100B_HUMAN      --------------MSELEKAMVALIDVFHQYSGREG-DKHKLKKSELKELINNELSHFL        45
sp|P29377|S100G_HUMAN      ---------------MSTKKSPEELKRIFEKYAAKEG-DPDQLSKDELKLLIQAEFPSLL        44
sp|P25815|S100P_HUMAN      --------------MTELETAMGMIIDVFSRYSGSEG-STQTLTKGELKVLMEKELPGFL        45
sp|Q8WXG8|S100Z_HUMAN      -------------MPTQLEMAMDTMIRIFHRYSGKER-KRFKLSKGELKLLLQRELTEFL        46
sp|Q5QJ38|TCHL1_HUMAN      --------------MPQLLRNVLCVIETFHKYASEDS-NGATLTGRELKQLIQGEFGDFF        45
sp|Q86YZ3|HORN_HUMAN       --------------MPKLLQGVITVIDVFYQYATQHG-EYDTLNKAELKELLENEFHQIL        45
                                                   :   :  .          :    :  ::  :

sp|P23297|S10A1_HUMAN      DAQ--KDVDAVDKVMKELDENGDGEVDFQEYVVLVAALTVACNNFFWENS----------        94
sp|P29034|S10A2_HUMAN      GEK--VDEEGLKKLMGSLDENSDQQVDFQEYAVFLALITVMCNDFFQGCPDRP-------        98
sp|P33764|S10A3_HUMAN      PTE--FRECDYNKFMSVLDTNKDCEVDFVEYVRSLACLCLYCHEYFKDCPSEPPCSQ---        101
sp|P26447|S10A4_HUMAN      GKR--TDEAAFQKLMSNLDSNRDNEVDFQEYCVFLSCIAMMCNEFFEGFPDKQPRKK---        101
sp|P33763|S10A5_HUMAN      GE---MKESSIDDLMKSLDKNSDQEIDFKEYSVFLTMLCMAYNDFFLEDNK---------        92
sp|P06703|S10A6_HUMAN      GSK--LQDAEIARLMEDLDRNKDQEVNFQEYVTFLGALALIYNEALKG------------        90
sp|P31151|S10A7_HUMAN      SACDKKGTNYLADVFEKKDKNEDKKIDFSEFLSLLGDIATDYHKQSHGAAPCSGGSQ---        101
sp|Q86SG5|S1A7A_HUMAN      SACDKKGIHYLATVFEKKDKNEDKKIDFSEFLSLLGDIAADYHKQSHGAAPCSGGSQ---        101
sp|Q5SY68|S1A7B_HUMAN      SGCEKSDMDYLSNALEKKDDNKDKKVNYSEFLSLLGDITIDHHKIMHGVAPCSGGSQ---        101
sp|P05109|S10A8_HUMAN      RKK------GADVWFKELDINTDGAVNFQEFLILVIKMGVAAHKKSHEESHKE-------        93
sp|P06702|S10A9_HUMAN      KKEN-KNEKVIEHIMEDLDTNADKQLSFEEFIMLMARLTWASHEKMHEGDEGPGHHHKP-        107
sp|P60903|S10AA_HUMAN      ENQ--KDPLAVDKIMKDLDQCRDGKVGFQSFFSLIAGLTIACNDYFVVHMKQKGKK----        97
sp|P31949|S10AB_HUMAN      KNQ--KDPGVLDRMMKKLDTNSDGQLDFSEFLNLIGGLAMACHDSFLKAVPSQKRT----        105
sp|P80511|S10AC_HUMAN      KNI--KDKAVIDEIFQGLDANQDEQVDFQEFISLVAIALKAAHYHTHKE-----------        92
sp|Q99584|S10AD_HUMAN      KD-----VGSLDEKMKSLDVNQDSELKFNEYWRLIGELAKEIRKKKDLKIRKK-------        98
sp|Q9HCY8|S10AE_HUMAN      PS-----NCGLEEKIANLGSCNDSKLEFRSFWELIGEAAKSVKLER------PVRGH---        104
sp|Q96FQ6|S10AG_HUMAN      SDT--GNRKAADKLIQNLDANHDGRISFDEYWTLIGGITGPIAKLIHEQEQQSSS-----        103
sp|P04271|S100B_HUMAN      EEI--KEQEVVDKVMETLDNDGDGECDFQEFMAFVAMVTTACHEFFEHE-----------        92
sp|P29377|S100G_HUMAN      KG-----PNTLDDLFQELDKNGDGEVSFEEFQVLVKKISQ--------------------        79
sp|P25815|S100P_HUMAN      QSG--KDKDAVDKLLKDLDANGDAQVDFSEFIVFVAAITSACHKYFEKAGLK--------        95
sp|Q8WXG8|S100Z_HUMAN      SCQ--KETQLVDKIVQDLDANKDNEVDFNEFVVMVAALTVACNDYFVEQLKKKGK-----        99
sp|Q5QJ38|TCHL1_HUMAN      QPC---VLHAVEKNSNLLNIDSNGIISFDEFVLAIFNLLNLCYLDIKSLLSSELRQVTKP        102
sp|Q86YZ3|HORN_HUMAN       KNP--NDPDTVDIILQSLDRDHNKKVDFTEYLLMIFKLVQARNKIIGKDYCQVSGSKLRD        103
                                             .   :    : .:   :

sp|P23297|S10A1_HUMAN      ------------------------------------------------------------        94
sp|P29034|S10A2_HUMAN      ------------------------------------------------------------        98
sp|P33764|S10A3_HUMAN      ------------------------------------------------------------        101
sp|P26447|S10A4_HUMAN      ------------------------------------------------------------        101
sp|P33763|S10A5_HUMAN      ------------------------------------------------------------        92
sp|P06703|S10A6_HUMAN      ------------------------------------------------------------        90
sp|P31151|S10A7_HUMAN      ------------------------------------------------------------        101
sp|Q86SG5|S1A7A_HUMAN      ------------------------------------------------------------        101
sp|Q5SY68|S1A7B_HUMAN      ------------------------------------------------------------        101
sp|P05109|S10A8_HUMAN      ------------------------------------------------------------        93
sp|P06702|S10A9_HUMAN      ------GLGEGTP-----------------------------------------------        114
```

```
sp|P60903|S10AA_HUMAN      ------------------------------------------------------------      97
sp|P31949|S10AB_HUMAN      ------------------------------------------------------------      105
sp|P80511|S10AC_HUMAN      ------------------------------------------------------------      92
sp|Q99584|S10AD_HUMAN      ------------------------------------------------------------      98
sp|Q9HCY8|S10AE_HUMAN      ------------------------------------------------------------      104
sp|Q96FQ6|S10AG_HUMAN      ------------------------------------------------------------      103
sp|P04271|S100B_HUMAN      ------------------------------------------------------------      92
sp|P29377|S100G_HUMAN      ------------------------------------------------------------      79
sp|P25815|S100P_HUMAN      ------------------------------------------------------------      95
sp|Q8WXG8|S100Z_HUMAN      ------------------------------------------------------------      99
sp|Q5QJ38|TCHL1_HUMAN      E----------KEKLDDVDVQATTGDGQWTVGTSPTQEKRML---PSGMASSSQLIPEES      149
sp|Q86YZ3|HORN_HUMAN       DTHQHQEEQEETEKEENKRQESSFSHSSWSAGENDSYSRNVRGSLKPGTESISRRLSFQR      163

sp|P23297|S10A1_HUMAN      ------------------------------------------------------------      94
sp|P29034|S10A2_HUMAN      ------------------------------------------------------------      98
sp|P33764|S10A3_HUMAN      ------------------------------------------------------------      101
sp|P26447|S10A4_HUMAN      ------------------------------------------------------------      101
sp|P33763|S10A5_HUMAN      ------------------------------------------------------------      92
sp|P06703|S10A6_HUMAN      ------------------------------------------------------------      90
sp|P31151|S10A7_HUMAN      ------------------------------------------------------------      101
sp|Q86SG5|S1A7A_HUMAN      ------------------------------------------------------------      101
sp|Q5SY68|S1A7B_HUMAN      ------------------------------------------------------------      101
sp|P05109|S10A8_HUMAN      ------------------------------------------------------------      93
sp|P06702|S10A9_HUMAN      ------------------------------------------------------------      114
sp|P60903|S10AA_HUMAN      ------------------------------------------------------------      97
sp|P31949|S10AB_HUMAN      ------------------------------------------------------------      105
sp|P80511|S10AC_HUMAN      ------------------------------------------------------------      92
sp|Q99584|S10AD_HUMAN      ------------------------------------------------------------      98
sp|Q9HCY8|S10AE_HUMAN      ------------------------------------------------------------      104
sp|Q96FQ6|S10AG_HUMAN      ------------------------------------------------------------      103
sp|P04271|S100B_HUMAN      ------------------------------------------------------------      92
sp|P29377|S100G_HUMAN      ------------------------------------------------------------      79
sp|P25815|S100P_HUMAN      ------------------------------------------------------------      95
sp|Q8WXG8|S100Z_HUMAN      ------------------------------------------------------------      99
sp|Q5QJ38|TCHL1_HUMAN      GAVGNNRVDPWREAKTHNFPGEASEHNDPKNK-H-LEGDEQSQE-VA--QDIQTTEDNEG      204
sp|Q86YZ3|HORN_HUMAN       DFS----------GQHNSYSGQSSSYGEQNSDSHQSSGRGQCGSGSGQSPNYGQHGSGSG      213

sp|P23297|S10A1_HUMAN      ------------------------------------------------------------      94
sp|P29034|S10A2_HUMAN      ------------------------------------------------------------      98
sp|P33764|S10A3_HUMAN      ------------------------------------------------------------      101
sp|P26447|S10A4_HUMAN      ------------------------------------------------------------      101
sp|P33763|S10A5_HUMAN      ------------------------------------------------------------      92
sp|P06703|S10A6_HUMAN      ------------------------------------------------------------      90
sp|P31151|S10A7_HUMAN      ------------------------------------------------------------      101
sp|Q86SG5|S1A7A_HUMAN      ------------------------------------------------------------      101
sp|Q5SY68|S1A7B_HUMAN      ------------------------------------------------------------      101
sp|P05109|S10A8_HUMAN      ------------------------------------------------------------      93
sp|P06702|S10A9_HUMAN      ------------------------------------------------------------      114
sp|P60903|S10AA_HUMAN      ------------------------------------------------------------      97
sp|P31949|S10AB_HUMAN      ------------------------------------------------------------      105
sp|P80511|S10AC_HUMAN      ------------------------------------------------------------      92
sp|Q99584|S10AD_HUMAN      ------------------------------------------------------------      98
sp|Q9HCY8|S10AE_HUMAN      ------------------------------------------------------------      104
sp|Q96FQ6|S10AG_HUMAN      ------------------------------------------------------------      103
sp|P04271|S100B_HUMAN      ------------------------------------------------------------      92
sp|P29377|S100G_HUMAN      ------------------------------------------------------------      79
sp|P25815|S100P_HUMAN      ------------------------------------------------------------      95
sp|Q8WXG8|S100Z_HUMAN      ------------------------------------------------------------      99
sp|Q5QJ38|TCHL1_HUMAN      QLKTNKPM-AGSKKTS---SPTERKGQDKEISQEGDEPAREQSVSKIRDQFGEQEGNLAT      260
sp|Q86YZ3|HORN_HUMAN       QSSSNDTHGSGSGQSSGFSQHKSSSGQSSGYSQHGSGSGH-------SSGYGQHG-SRSG      265

sp|P23297|S10A1_HUMAN      ------------------------------------------------------------      94
sp|P29034|S10A2_HUMAN      ------------------------------------------------------------      98
sp|P33764|S10A3_HUMAN      ------------------------------------------------------------      101
```

```
sp|P26447|S10A4_HUMAN      ------------------------------------------------------------      101
sp|P33763|S10A5_HUMAN      ------------------------------------------------------------      92
sp|P06703|S10A6_HUMAN      ------------------------------------------------------------      90
sp|P31151|S10A7_HUMAN      ------------------------------------------------------------      101
sp|Q86SG5|S1A7A_HUMAN      ------------------------------------------------------------      101
sp|Q5SY68|S1A7B_HUMAN      ------------------------------------------------------------      101
sp|P05109|S10A8_HUMAN      ------------------------------------------------------------      93
sp|P06702|S10A9_HUMAN      ------------------------------------------------------------      114
sp|P60903|S10AA_HUMAN      ------------------------------------------------------------      97
sp|P31949|S10AB_HUMAN      ------------------------------------------------------------      105
sp|P80511|S10AC_HUMAN      ------------------------------------------------------------      92
sp|Q99584|S10AD_HUMAN      ------------------------------------------------------------      98
sp|Q9HCY8|S10AE_HUMAN      ------------------------------------------------------------      104
sp|Q96FQ6|S10AG_HUMAN      ------------------------------------------------------------      103
sp|P04271|S100B_HUMAN      ------------------------------------------------------------      92
sp|P29377|S100G_HUMAN      ------------------------------------------------------------      79
sp|P25815|S100P_HUMAN      ------------------------------------------------------------      95
sp|Q8WXG8|S100Z_HUMAN      ------------------------------------------------------------      99
sp|Q5QJ38|TCHL1_HUMAN      QSSPPKEATQRPC-------------EDQEVRTEKEKHSNIQEPPLQREDEPSSQHADLP      307
sp|Q86YZ3|HORN_HUMAN       QSSRG--ERHRSSSGSSSSYGQHGSGSRQSLGHGRQGSGSRQSPSHVRHGS-GSGHSSSH      322

sp|P23297|S10A1_HUMAN      ------------------------------------------------------------      94
sp|P29034|S10A2_HUMAN      ------------------------------------------------------------      98
sp|P33764|S10A3_HUMAN      ------------------------------------------------------------      101
sp|P26447|S10A4_HUMAN      ------------------------------------------------------------      101
sp|P33763|S10A5_HUMAN      ------------------------------------------------------------      92
sp|P06703|S10A6_HUMAN      ------------------------------------------------------------      90
sp|P31151|S10A7_HUMAN      ------------------------------------------------------------      101
sp|Q86SG5|S1A7A_HUMAN      ------------------------------------------------------------      101
sp|Q5SY68|S1A7B_HUMAN      ------------------------------------------------------------      101
sp|P05109|S10A8_HUMAN      ------------------------------------------------------------      93
sp|P06702|S10A9_HUMAN      ------------------------------------------------------------      114
sp|P60903|S10AA_HUMAN      ------------------------------------------------------------      97
sp|P31949|S10AB_HUMAN      ------------------------------------------------------------      105
sp|P80511|S10AC_HUMAN      ------------------------------------------------------------      92
sp|Q99584|S10AD_HUMAN      ------------------------------------------------------------      98
sp|Q9HCY8|S10AE_HUMAN      ------------------------------------------------------------      104
sp|Q96FQ6|S10AG_HUMAN      ------------------------------------------------------------      103
sp|P04271|S100B_HUMAN      ------------------------------------------------------------      92
sp|P29377|S100G_HUMAN      ------------------------------------------------------------      79
sp|P25815|S100P_HUMAN      ------------------------------------------------------------      95
sp|Q8WXG8|S100Z_HUMAN      ------------------------------------------------------------      99
sp|Q5QJ38|TCHL1_HUMAN      EQAAARS-PSQTQKS-TDSKDVCRMFDTQEPGKDADQTPAKTKNLGEPEDYGRTSETQ--      363
sp|Q86YZ3|HORN_HUMAN       GQHGSGSSYSYSRGHYESGSGQTSGFGQHESGS------------GQSSGYSKHGSGSGH      370

sp|P23297|S10A1_HUMAN      ------------------------------------------------------------      94
sp|P29034|S10A2_HUMAN      ------------------------------------------------------------      98
sp|P33764|S10A3_HUMAN      ------------------------------------------------------------      101
sp|P26447|S10A4_HUMAN      ------------------------------------------------------------      101
sp|P33763|S10A5_HUMAN      ------------------------------------------------------------      92
sp|P06703|S10A6_HUMAN      ------------------------------------------------------------      90
sp|P31151|S10A7_HUMAN      ------------------------------------------------------------      101
sp|Q86SG5|S1A7A_HUMAN      ------------------------------------------------------------      101
sp|Q5SY68|S1A7B_HUMAN      ------------------------------------------------------------      101
sp|P05109|S10A8_HUMAN      ------------------------------------------------------------      93
sp|P06702|S10A9_HUMAN      ------------------------------------------------------------      114
sp|P60903|S10AA_HUMAN      ------------------------------------------------------------      97
sp|P31949|S10AB_HUMAN      ------------------------------------------------------------      105
sp|P80511|S10AC_HUMAN      ------------------------------------------------------------      92
sp|Q99584|S10AD_HUMAN      ------------------------------------------------------------      98
sp|Q9HCY8|S10AE_HUMAN      ------------------------------------------------------------      104
sp|Q96FQ6|S10AG_HUMAN      ------------------------------------------------------------      103
sp|P04271|S100B_HUMAN      ------------------------------------------------------------      92
sp|P29377|S100G_HUMAN      ------------------------------------------------------------      79
sp|P25815|S100P_HUMAN      ------------------------------------------------------------      95
```

```
sp|Q8WXG8|S100Z_HUMAN      ------------------------------------------------------------      99
sp|Q5QJ38|TCHL1_HUMAN      ----EKE-------------CETKDLPVQYGSRNGSETSDMRDERKERRGPEAHGT----      402
sp|Q86YZ3|HORN_HUMAN       SSSQGQHGSTSGQASSSGQHGSSSRQSSSYGQHESA----SR--HSSGRGQHSSGSGQSP      424

sp|P23297|S10A1_HUMAN      ------------------------------------------------------------      94
sp|P29034|S10A2_HUMAN      ------------------------------------------------------------      98
sp|P33764|S10A3_HUMAN      ------------------------------------------------------------      101
sp|P26447|S10A4_HUMAN      ------------------------------------------------------------      101
sp|P33763|S10A5_HUMAN      ------------------------------------------------------------      92
sp|P06703|S10A6_HUMAN      ------------------------------------------------------------      90
sp|P31151|S10A7_HUMAN      ------------------------------------------------------------      101
sp|Q86SG5|S1A7A_HUMAN      ------------------------------------------------------------      101
sp|Q5SY68|S1A7B_HUMAN      ------------------------------------------------------------      101
sp|P05109|S10A8_HUMAN      ------------------------------------------------------------      93
sp|P06702|S10A9_HUMAN      ------------------------------------------------------------      114
sp|P60903|S10AA_HUMAN      ------------------------------------------------------------      97
sp|P31949|S10AB_HUMAN      ------------------------------------------------------------      105
sp|P80511|S10AC_HUMAN      ------------------------------------------------------------      92
sp|Q99584|S10AD_HUMAN      ------------------------------------------------------------      98
sp|Q9HCY8|S10AE_HUMAN      ------------------------------------------------------------      104
sp|Q96FQ6|S10AG_HUMAN      ------------------------------------------------------------      103
sp|P04271|S100B_HUMAN      ------------------------------------------------------------      92
sp|P29377|S100G_HUMAN      ------------------------------------------------------------      79
sp|P25815|S100P_HUMAN      ------------------------------------------------------------      95
sp|Q8WXG8|S100Z_HUMAN      ------------------------------------------------------------      99
sp|Q5QJ38|TCHL1_HUMAN      -AGQKERDRKTRPL------VLETQTQDGKYQELQGLSKSKDAEK---GSETQYLSSEGG      452
sp|Q86YZ3|HORN_HUMAN       GHGQRGSGSGQSPSSGQHGTGFGRSSSSGPYVSGSGYSSGFGHHESSSEHSSGYTQHGSG      484

sp|P23297|S10A1_HUMAN      ------------------------------------------------------------      94
sp|P29034|S10A2_HUMAN      ------------------------------------------------------------      98
sp|P33764|S10A3_HUMAN      ------------------------------------------------------------      101
sp|P26447|S10A4_HUMAN      ------------------------------------------------------------      101
sp|P33763|S10A5_HUMAN      ------------------------------------------------------------      92
sp|P06703|S10A6_HUMAN      ------------------------------------------------------------      90
sp|P31151|S10A7_HUMAN      ------------------------------------------------------------      101
sp|Q86SG5|S1A7A_HUMAN      ------------------------------------------------------------      101
sp|Q5SY68|S1A7B_HUMAN      ------------------------------------------------------------      101
sp|P05109|S10A8_HUMAN      ------------------------------------------------------------      93
sp|P06702|S10A9_HUMAN      ------------------------------------------------------------      114
sp|P60903|S10AA_HUMAN      ------------------------------------------------------------      97
sp|P31949|S10AB_HUMAN      ------------------------------------------------------------      105
sp|P80511|S10AC_HUMAN      ------------------------------------------------------------      92
sp|Q99584|S10AD_HUMAN      ------------------------------------------------------------      98
sp|Q9HCY8|S10AE_HUMAN      ------------------------------------------------------------      104
sp|Q96FQ6|S10AG_HUMAN      ------------------------------------------------------------      103
sp|P04271|S100B_HUMAN      ------------------------------------------------------------      92
sp|P29377|S100G_HUMAN      ------------------------------------------------------------      79
sp|P25815|S100P_HUMAN      ------------------------------------------------------------      95
sp|Q8WXG8|S100Z_HUMAN      ------------------------------------------------------------      99
sp|Q5QJ38|TCHL1_HUMAN      ------DQTHPELEGTAVSGEEAEHTKEGTAEAFVNSKNAPAAERTLGARERTQDLAPLE      506
sp|Q86YZ3|HORN_HUMAN       SGHSSGHGQHGSRSGQSSRGERQ-GSSAGSSSSYGQ--HGSGSRQSLGHSRHGSGSGQSP      541

sp|P23297|S10A1_HUMAN      ------------------------------------------------------------      94
sp|P29034|S10A2_HUMAN      ------------------------------------------------------------      98
sp|P33764|S10A3_HUMAN      ------------------------------------------------------------      101
sp|P26447|S10A4_HUMAN      ------------------------------------------------------------      101
sp|P33763|S10A5_HUMAN      ------------------------------------------------------------      92
sp|P06703|S10A6_HUMAN      ------------------------------------------------------------      90
sp|P31151|S10A7_HUMAN      ------------------------------------------------------------      101
sp|Q86SG5|S1A7A_HUMAN      ------------------------------------------------------------      101
sp|Q5SY68|S1A7B_HUMAN      ------------------------------------------------------------      101
sp|P05109|S10A8_HUMAN      ------------------------------------------------------------      93
sp|P06702|S10A9_HUMAN      ------------------------------------------------------------      114
sp|P60903|S10AA_HUMAN      ------------------------------------------------------------      97
```

```
sp|P31949|S10AB_HUMAN      ------------------------------------------------------------      105
sp|P80511|S10AC_HUMAN      ------------------------------------------------------------      92
sp|Q99584|S10AD_HUMAN      ------------------------------------------------------------      98
sp|Q9HCY8|S10AE_HUMAN      ------------------------------------------------------------      104
sp|Q96FQ6|S10AG_HUMAN      ------------------------------------------------------------      103
sp|P04271|S100B_HUMAN      ------------------------------------------------------------      92
sp|P29377|S100G_HUMAN      ------------------------------------------------------------      79
sp|P25815|S100P_HUMAN      ------------------------------------------------------------      95
sp|Q8WXG8|S100Z_HUMAN      ------------------------------------------------------------      99
sp|Q5QJ38|TCHL1_HUMAN      KQSVGENTRVTKTHDQPVEEEDGYQGE--DPESPFTQSDEG-----SSETPNSLASEEGN      559
sp|Q86YZ3|HORN_HUMAN       SPSRGRHES--GSRQSSSYGPHGYGSGRSSSRGPYESGSGHSSGLGHQESRSGQSSGYGQ      599


sp|P23297|S10A1_HUMAN      ------------------------------------------------------------      94
sp|P29034|S10A2_HUMAN      ------------------------------------------------------------      98
sp|P33764|S10A3_HUMAN      ------------------------------------------------------------      101
sp|P26447|S10A4_HUMAN      ------------------------------------------------------------      101
sp|P33763|S10A5_HUMAN      ------------------------------------------------------------      92
sp|P06703|S10A6_HUMAN      ------------------------------------------------------------      90
sp|P31151|S10A7_HUMAN      ------------------------------------------------------------      101
sp|Q86SG5|S1A7A_HUMAN      ------------------------------------------------------------      101
sp|Q5SY68|S1A7B_HUMAN      ------------------------------------------------------------      101
sp|P05109|S10A8_HUMAN      ------------------------------------------------------------      93
sp|P06702|S10A9_HUMAN      ------------------------------------------------------------      114
sp|P60903|S10AA_HUMAN      ------------------------------------------------------------      97
sp|P31949|S10AB_HUMAN      ------------------------------------------------------------      105
sp|P80511|S10AC_HUMAN      ------------------------------------------------------------      92
sp|Q99584|S10AD_HUMAN      ------------------------------------------------------------      98
sp|Q9HCY8|S10AE_HUMAN      ------------------------------------------------------------      104
sp|Q96FQ6|S10AG_HUMAN      ------------------------------------------------------------      103
sp|P04271|S100B_HUMAN      ------------------------------------------------------------      92
sp|P29377|S100G_HUMAN      ------------------------------------------------------------      79
sp|P25815|S100P_HUMAN      ------------------------------------------------------------      95
sp|Q8WXG8|S100Z_HUMAN      ------------------------------------------------------------      99
sp|Q5QJ38|TCHL1_HUMAN      SSSETGELPVQGDSQSQGDQHGESVQGGHNNNPD--TQRQGTPGEKNRALEAVVPAVRGE      617
sp|Q86YZ3|HORN_HUMAN       HGSSSGHSSTHGQH---GSTSGQSSSCGQHGATSGQSSSHGQHGSGSSQ-----SSRYG-      650


sp|P23297|S10A1_HUMAN      ------------------------------------------------------------      94
sp|P29034|S10A2_HUMAN      ------------------------------------------------------------      98
sp|P33764|S10A3_HUMAN      ------------------------------------------------------------      101
sp|P26447|S10A4_HUMAN      ------------------------------------------------------------      101
sp|P33763|S10A5_HUMAN      ------------------------------------------------------------      92
sp|P06703|S10A6_HUMAN      ------------------------------------------------------------      90
sp|P31151|S10A7_HUMAN      ------------------------------------------------------------      101
sp|Q86SG5|S1A7A_HUMAN      ------------------------------------------------------------      101
sp|Q5SY68|S1A7B_HUMAN      ------------------------------------------------------------      101
sp|P05109|S10A8_HUMAN      ------------------------------------------------------------      93
sp|P06702|S10A9_HUMAN      ------------------------------------------------------------      114
sp|P60903|S10AA_HUMAN      ------------------------------------------------------------      97
sp|P31949|S10AB_HUMAN      ------------------------------------------------------------      105
sp|P80511|S10AC_HUMAN      ------------------------------------------------------------      92
sp|Q99584|S10AD_HUMAN      ------------------------------------------------------------      98
sp|Q9HCY8|S10AE_HUMAN      ------------------------------------------------------------      104
sp|Q96FQ6|S10AG_HUMAN      ------------------------------------------------------------      103
sp|P04271|S100B_HUMAN      ------------------------------------------------------------      92
sp|P29377|S100G_HUMAN      ------------------------------------------------------------      79
sp|P25815|S100P_HUMAN      ------------------------------------------------------------      95
sp|Q8WXG8|S100Z_HUMAN      ------------------------------------------------------------      99
sp|Q5QJ38|TCHL1_HUMAN      DVQLTEDQEQPARGEHKNQ-----GPGTKGPGAAV----EPNGHPEAQESTAGDENRKSL      668
sp|Q86YZ3|HORN_HUMAN       -QQGSGSGQSPSRGRHGSDFGHSSSYGQHGSGSGWSSSNGPHGSVSGQSS----------      699


sp|P23297|S10A1_HUMAN      ------------------------------------------------------------      94
sp|P29034|S10A2_HUMAN      ------------------------------------------------------------      98
sp|P33764|S10A3_HUMAN      ------------------------------------------------------------      101
sp|P26447|S10A4_HUMAN      ------------------------------------------------------------      101
```

```
sp|P33763|S10A5_HUMAN      ------------------------------------------------------------        92
sp|P06703|S10A6_HUMAN      ------------------------------------------------------------        90
sp|P31151|S10A7_HUMAN      ------------------------------------------------------------        101
sp|Q86SG5|S1A7A_HUMAN      ------------------------------------------------------------        101
sp|Q5SY68|S1A7B_HUMAN      ------------------------------------------------------------        101
sp|P05109|S10A8_HUMAN      ------------------------------------------------------------        93
sp|P06702|S10A9_HUMAN      ------------------------------------------------------------        114
sp|P60903|S10AA_HUMAN      ------------------------------------------------------------        97
sp|P31949|S10AB_HUMAN      ------------------------------------------------------------        105
sp|P80511|S10AC_HUMAN      ------------------------------------------------------------        92
sp|Q99584|S10AD_HUMAN      ------------------------------------------------------------        98
sp|Q9HCY8|S10AE_HUMAN      ------------------------------------------------------------        104
sp|Q96FQ6|S10AG_HUMAN      ------------------------------------------------------------        103
sp|P04271|S100B_HUMAN      ------------------------------------------------------------        92
sp|P29377|S100G_HUMAN      ------------------------------------------------------------        79
sp|P25815|S100P_HUMAN      ------------------------------------------------------------        95
sp|Q8WXG8|S100Z_HUMAN      ------------------------------------------------------------        99
sp|Q5QJ38|TCHL1_HUMAN      EIEITGALDEDFTDQLSLMQLPGKGDSRNELKVQGPSSKEEKGRATEAQNTLLESLDED-        727
sp|Q86YZ3|HORN_HUMAN       ----------GFGH------KSGSGQSSG-YSQHGSGSSHSSGYRKHGSRSGQSSRSEQH        742

sp|P23297|S10A1_HUMAN      ------------------------------------------------------------        94
sp|P29034|S10A2_HUMAN      ------------------------------------------------------------        98
sp|P33764|S10A3_HUMAN      ------------------------------------------------------------        101
sp|P26447|S10A4_HUMAN      ------------------------------------------------------------        101
sp|P33763|S10A5_HUMAN      ------------------------------------------------------------        92
sp|P06703|S10A6_HUMAN      ------------------------------------------------------------        90
sp|P31151|S10A7_HUMAN      ------------------------------------------------------------        101
sp|Q86SG5|S1A7A_HUMAN      ------------------------------------------------------------        101
sp|Q5SY68|S1A7B_HUMAN      ------------------------------------------------------------        101
sp|P05109|S10A8_HUMAN      ------------------------------------------------------------        93
sp|P06702|S10A9_HUMAN      ------------------------------------------------------------        114
sp|P60903|S10AA_HUMAN      ------------------------------------------------------------        97
sp|P31949|S10AB_HUMAN      ------------------------------------------------------------        105
sp|P80511|S10AC_HUMAN      ------------------------------------------------------------        92
sp|Q99584|S10AD_HUMAN      ------------------------------------------------------------        98
sp|Q9HCY8|S10AE_HUMAN      ------------------------------------------------------------        104
sp|Q96FQ6|S10AG_HUMAN      ------------------------------------------------------------        103
sp|P04271|S100B_HUMAN      ------------------------------------------------------------        92
sp|P29377|S100G_HUMAN      ------------------------------------------------------------        79
sp|P25815|S100P_HUMAN      ------------------------------------------------------------        95
sp|Q8WXG8|S100Z_HUMAN      ------------------------------------------------------------        99
sp|Q5QJ38|TCHL1_HUMAN      -NSASLKIQLETKEPVTSEEEDESPQELAGEGGDQKSPAKKEHNSSVPWSSLEKQMQRDQ        786
sp|Q86YZ3|HORN_HUMAN       GSSSGLSSSYGQHGS---GSHQSSGHGRQG-SGSGHSPSRVRHGSSSGHSSSHGQ-HGSG        797

sp|P23297|S10A1_HUMAN      ------------------------------------------------------------        94
sp|P29034|S10A2_HUMAN      ------------------------------------------------------------        98
sp|P33764|S10A3_HUMAN      ------------------------------------------------------------        101
sp|P26447|S10A4_HUMAN      ------------------------------------------------------------        101
sp|P33763|S10A5_HUMAN      ------------------------------------------------------------        92
sp|P06703|S10A6_HUMAN      ------------------------------------------------------------        90
sp|P31151|S10A7_HUMAN      ------------------------------------------------------------        101
sp|Q86SG5|S1A7A_HUMAN      ------------------------------------------------------------        101
sp|Q5SY68|S1A7B_HUMAN      ------------------------------------------------------------        101
sp|P05109|S10A8_HUMAN      ------------------------------------------------------------        93
sp|P06702|S10A9_HUMAN      ------------------------------------------------------------        114
sp|P60903|S10AA_HUMAN      ------------------------------------------------------------        97
sp|P31949|S10AB_HUMAN      ------------------------------------------------------------        105
sp|P80511|S10AC_HUMAN      ------------------------------------------------------------        92
sp|Q99584|S10AD_HUMAN      ------------------------------------------------------------        98
sp|Q9HCY8|S10AE_HUMAN      ------------------------------------------------------------        104
sp|Q96FQ6|S10AG_HUMAN      ------------------------------------------------------------        103
sp|P04271|S100B_HUMAN      ------------------------------------------------------------        92
sp|P29377|S100G_HUMAN      ------------------------------------------------------------        79
sp|P25815|S100P_HUMAN      ------------------------------------------------------------        95
sp|Q8WXG8|S100Z_HUMAN      ------------------------------------------------------------        99
```

```
sp|Q5QJ38|TCHL1_HUMAN      EPCSVERGAVYSSPLYQYLQEKILQQTNVTQEEHQKQVQIAQASGPELCSVSLTSEISDC        846
sp|Q86YZ3|HORN_HUMAN       TSCS-----------------------------------------------------SSC        804


sp|P23297|S10A1_HUMAN      ------------------------------------------------------------        94
sp|P29034|S10A2_HUMAN      ------------------------------------------------------------        98
sp|P33764|S10A3_HUMAN      ------------------------------------------------------------        101
sp|P26447|S10A4_HUMAN      ------------------------------------------------------------        101
sp|P33763|S10A5_HUMAN      ------------------------------------------------------------        92
sp|P06703|S10A6_HUMAN      ------------------------------------------------------------        90
sp|P31151|S10A7_HUMAN      ------------------------------------------------------------        101
sp|Q86SG5|S1A7A_HUMAN      ------------------------------------------------------------        101
sp|Q5SY68|S1A7B_HUMAN      ------------------------------------------------------------        101
sp|P05109|S10A8_HUMAN      ------------------------------------------------------------        93
sp|P06702|S10A9_HUMAN      ------------------------------------------------------------        114
sp|P60903|S10AA_HUMAN      ------------------------------------------------------------        97
sp|P31949|S10AB_HUMAN      ------------------------------------------------------------        105
sp|P80511|S10AC_HUMAN      ------------------------------------------------------------        92
sp|Q99584|S10AD_HUMAN      ------------------------------------------------------------        98
sp|Q9HCY8|S10AE_HUMAN      ------------------------------------------------------------        104
sp|Q96FQ6|S10AG_HUMAN      ------------------------------------------------------------        103
sp|P04271|S100B_HUMAN      ------------------------------------------------------------        92
sp|P29377|S100G_HUMAN      ------------------------------------------------------------        79
sp|P25815|S100P_HUMAN      ------------------------------------------------------------        95
sp|Q8WXG8|S100Z_HUMAN      ------------------------------------------------------------        99
sp|Q5QJ38|TCHL1 HUMAN      SVFFNYSQASQPY-------TRGLPLDESPAGA-------------------QETPAPQA        880
sp|Q86YZ3|HORN_HUMAN       GHYESGSGQASGFGQHESGSGQGYSQHGSASGHFSSQGRHGSTSGQSSSSGQHDSSSGQS        864


sp|P23297|S10A1_HUMAN      ------------------------------------------------------------        94
sp|P29034|S10A2_HUMAN      ------------------------------------------------------------        98
sp|P33764|S10A3_HUMAN      ------------------------------------------------------------        101
sp|P26447|S10A4_HUMAN      ------------------------------------------------------------        101
sp|P33763|S10A5_HUMAN      ------------------------------------------------------------        92
sp|P06703|S10A6_HUMAN      ------------------------------------------------------------        90
sp|P31151|S10A7_HUMAN      ------------------------------------------------------------        101
sp|Q86SG5|S1A7A_HUMAN      ------------------------------------------------------------        101
sp|Q5SY68|S1A7B_HUMAN      ------------------------------------------------------------        101
sp|P05109|S10A8_HUMAN      ------------------------------------------------------------        93
sp|P06702|S10A9_HUMAN      ------------------------------------------------------------        114
sp|P60903|S10AA_HUMAN      ------------------------------------------------------------        97
sp|P31949|S10AB_HUMAN      ------------------------------------------------------------        105
sp|P80511|S10AC_HUMAN      ------------------------------------------------------------        92
sp|Q99584|S10AD_HUMAN      ------------------------------------------------------------        98
sp|Q9HCY8|S10AE_HUMAN      ------------------------------------------------------------        104
sp|Q96FQ6|S10AG_HUMAN      ------------------------------------------------------------        103
sp|P04271|S100B_HUMAN      ------------------------------------------------------------        92
sp|P29377|S100G_HUMAN      ------------------------------------------------------------        79
sp|P25815|S100P_HUMAN      ------------------------------------------------------------        95
sp|Q8WXG8|S100Z_HUMAN      ------------------------------------------------------------        99
sp|Q5QJ38|TCHL1_HUMAN      L----------------------EDKQGHPQRERL-----VLQREASTT-------KQ--        904
sp|Q86YZ3|HORN HUMAN       SSYGQHESASHHASGRGRHGSGSGQSPGHGQRGSGSGQSPSYGRHGSGSGRSSSSGRHGS        924


sp|P23297|S10A1_HUMAN      ------------------------------------------------------------        94
sp|P29034|S10A2_HUMAN      ------------------------------------------------------------        98
sp|P33764|S10A3_HUMAN      ------------------------------------------------------------        101
sp|P26447|S10A4_HUMAN      ------------------------------------------------------------        101
sp|P33763|S10A5_HUMAN      ------------------------------------------------------------        92
sp|P06703|S10A6_HUMAN      ------------------------------------------------------------        90
sp|P31151|S10A7_HUMAN      ------------------------------------------------------------        101
sp|Q86SG5|S1A7A_HUMAN      ------------------------------------------------------------        101
sp|Q5SY68|S1A7B_HUMAN      ------------------------------------------------------------        101
sp|P05109|S10A8_HUMAN      ------------------------------------------------------------        93
sp|P06702|S10A9_HUMAN      ------------------------------------------------------------        114
sp|P60903|S10AA_HUMAN      ------------------------------------------------------------        97
sp|P31949|S10AB_HUMAN      ------------------------------------------------------------        105
```

```
sp|P80511|S10AC_HUMAN      ------------------------------------------------------------      92
sp|Q99584|S10AD_HUMAN      ------------------------------------------------------------      98
sp|Q9HCY8|S10AE_HUMAN      ------------------------------------------------------------      104
sp|Q96FQ6|S10AG_HUMAN      ------------------------------------------------------------      103
sp|P04271|S100B_HUMAN      ------------------------------------------------------------      92
sp|P29377|S100G_HUMAN      ------------------------------------------------------------      79
sp|P25815|S100P_HUMAN      ------------------------------------------------------------      95
sp|Q8WXG8|S100Z_HUMAN      ------------------------------------------------------------      99
sp|Q5QJ38|TCHL1_HUMAN      ------------------------------------------------------------      904
sp|Q86YZ3|HORN_HUMAN       GSGQSSGFGHKSSSGQSSGYTQHGSGSGHSSSYEQHGSRSGQSSRSEQHGSSSGSSSSYG      984

sp|P23297|S10A1_HUMAN      ------------------------------------------------------------      94
sp|P29034|S10A2_HUMAN      ------------------------------------------------------------      98
sp|P33764|S10A3_HUMAN      ------------------------------------------------------------      101
sp|P26447|S10A4_HUMAN      ------------------------------------------------------------      101
sp|P33763|S10A5_HUMAN      ------------------------------------------------------------      92
sp|P06703|S10A6_HUMAN      ------------------------------------------------------------      90
sp|P31151|S10A7_HUMAN      ------------------------------------------------------------      101
sp|Q86SG5|S1A7A_HUMAN      ------------------------------------------------------------      101
sp|Q5SY68|S1A7B_HUMAN      ------------------------------------------------------------      101
sp|P05109|S10A8_HUMAN      ------------------------------------------------------------      93
sp|P06702|S10A9_HUMAN      ------------------------------------------------------------      114
sp|P60903|S10AA_HUMAN      ------------------------------------------------------------      97
sp|P31949|S10AB_HUMAN      ------------------------------------------------------------      105
sp|P80511|S10AC_HUMAN      ------------------------------------------------------------      92
sp|Q99584|S10AD_HUMAN      ------------------------------------------------------------      98
sp|Q9HCY8|S10AE_HUMAN      ------------------------------------------------------------      104
sp|Q96FQ6|S10AG_HUMAN      ------------------------------------------------------------      103
sp|P04271|S100B_HUMAN      ------------------------------------------------------------      92
sp|P29377|S100G_HUMAN      ------------------------------------------------------------      79
sp|P25815|S100P_HUMAN      ------------------------------------------------------------      95
sp|Q8WXG8|S100Z_HUMAN      ------------------------------------------------------------      99
sp|Q5QJ38|TCHL1_HUMAN      ------------------------------------------------------------      904
sp|Q86YZ3|HORN_HUMAN       QHGSGSRQSLGHGQHGSGSGQSPSPSRGRHGSGSGQSSSYGPYRSGSGWSSSRGPYESGS      1044

sp|P23297|S10A1_HUMAN      ------------------------------------------------------------      94
sp|P29034|S10A2_HUMAN      ------------------------------------------------------------      98
sp|P33764|S10A3_HUMAN      ------------------------------------------------------------      101
sp|P26447|S10A4_HUMAN      ------------------------------------------------------------      101
sp|P33763|S10A5_HUMAN      ------------------------------------------------------------      92
sp|P06703|S10A6_HUMAN      ------------------------------------------------------------      90
sp|P31151|S10A7_HUMAN      ------------------------------------------------------------      101
sp|Q86SG5|S1A7A_HUMAN      ------------------------------------------------------------      101
sp|Q5SY68|S1A7B_HUMAN      ------------------------------------------------------------      101
sp|P05109|S10A8_HUMAN      ------------------------------------------------------------      93
sp|P06702|S10A9_HUMAN      ------------------------------------------------------------      114
sp|P60903|S10AA_HUMAN      ------------------------------------------------------------      97
sp|P31949|S10AB_HUMAN      ------------------------------------------------------------      105
sp|P80511|S10AC_HUMAN      ------------------------------------------------------------      92
sp|Q99584|S10AD_HUMAN      ------------------------------------------------------------      98
sp|Q9HCY8|S10AE_HUMAN      ------------------------------------------------------------      104
sp|Q96FQ6|S10AG_HUMAN      ------------------------------------------------------------      103
sp|P04271|S100B_HUMAN      ------------------------------------------------------------      92
sp|P29377|S100G_HUMAN      ------------------------------------------------------------      79
sp|P25815|S100P_HUMAN      ------------------------------------------------------------      95
sp|Q8WXG8|S100Z_HUMAN      ------------------------------------------------------------      99
sp|Q5QJ38|TCHL1_HUMAN      ------------------------------------------------------------      904
sp|Q86YZ3|HORN_HUMAN       GHSSGLGHRESRSGQSSGYGQHGSSSGHSSTHGQHGSTSGQSSSCGQHGASSGQSSSHGQ      1104

sp|P23297|S10A1_HUMAN      ------------------------------------------------------------      94
sp|P29034|S10A2_HUMAN      ------------------------------------------------------------      98
sp|P33764|S10A3_HUMAN      ------------------------------------------------------------      101
sp|P26447|S10A4_HUMAN      ------------------------------------------------------------      101
sp|P33763|S10A5_HUMAN      ------------------------------------------------------------      92
```

```
sp|P06703|S10A6_HUMAN      ------------------------------------------------------------      90
sp|P31151|S10A7_HUMAN      ------------------------------------------------------------      101
sp|Q86SG5|S1A7A_HUMAN      ------------------------------------------------------------      101
sp|Q5SY68|S1A7B_HUMAN      ------------------------------------------------------------      101
sp|P05109|S10A8_HUMAN      ------------------------------------------------------------      93
sp|P06702|S10A9_HUMAN      ------------------------------------------------------------      114
sp|P60903|S10AA_HUMAN      ------------------------------------------------------------      97
sp|P31949|S10AB_HUMAN      ------------------------------------------------------------      105
sp|P80511|S10AC_HUMAN      ------------------------------------------------------------      92
sp|Q99584|S10AD_HUMAN      ------------------------------------------------------------      98
sp|Q9HCY8|S10AE_HUMAN      ------------------------------------------------------------      104
sp|Q96FQ6|S10AG_HUMAN      ------------------------------------------------------------      103
sp|P04271|S100B_HUMAN      ------------------------------------------------------------      92
sp|P29377|S100G_HUMAN      ------------------------------------------------------------      79
sp|P25815|S100P_HUMAN      ------------------------------------------------------------      95
sp|Q8WXG8|S100Z_HUMAN      ------------------------------------------------------------      99
sp|Q5QJ38|TCHL1_HUMAN      ------------------------------------------------------------      904
sp|Q86YZ3|HORN_HUMAN       HGSGSSQSSGYGRQGSGSGQSPGHGQRGSGSRQSPSYGRHGSGSGRSSSSGQHGSGLGES      1164

sp|P23297|S10A1_HUMAN      ------------------------------------------------------------      94
sp|P29034|S10A2_HUMAN      ------------------------------------------------------------      98
sp|P33764|S10A3_HUMAN      ------------------------------------------------------------      101
sp|P26447|S10A4_HUMAN      ------------------------------------------------------------      101
sp|P33763|S10A5_HUMAN      ------------------------------------------------------------      92
sp|P06703|S10A6_HUMAN      ------------------------------------------------------------      90
sp|P31151|S10A7_HUMAN      ------------------------------------------------------------      101
sp|Q86SG5|S1A7A_HUMAN      ------------------------------------------------------------      101
sp|Q5SY68|S1A7B_HUMAN      ------------------------------------------------------------      101
sp|P05109|S10A8_HUMAN      ------------------------------------------------------------      93
sp|P06702|S10A9_HUMAN      ------------------------------------------------------------      114
sp|P60903|S10AA_HUMAN      ------------------------------------------------------------      97
sp|P31949|S10AB_HUMAN      ------------------------------------------------------------      105
sp|P80511|S10AC_HUMAN      ------------------------------------------------------------      92
sp|Q99584|S10AD_HUMAN      ------------------------------------------------------------      98
sp|Q9HCY8|S10AE_HUMAN      ------------------------------------------------------------      104
sp|Q96FQ6|S10AG_HUMAN      ------------------------------------------------------------      103
sp|P04271|S100B_HUMAN      ------------------------------------------------------------      92
sp|P29377|S100G_HUMAN      ------------------------------------------------------------      79
sp|P25815|S100P_HUMAN      ------------------------------------------------------------      95
sp|Q8WXG8|S100Z_HUMAN      ------------------------------------------------------------      99
sp|Q5QJ38|TCHL1_HUMAN      ------------------------------------------------------------      904
sp|Q86YZ3|HORN_HUMAN       SGFGHHESSSGQSSSYSQHGSGSGHSSGYGQHGSRSGQSSRGERHGSSSGSSSHYGQHGS      1224

sp|P23297|S10A1_HUMAN      ------------------------------------------------------------      94
sp|P29034|S10A2_HUMAN      ------------------------------------------------------------      98
sp|P33764|S10A3_HUMAN      ------------------------------------------------------------      101
sp|P26447|S10A4_HUMAN      ------------------------------------------------------------      101
sp|P33763|S10A5_HUMAN      ------------------------------------------------------------      92
sp|P06703|S10A6_HUMAN      ------------------------------------------------------------      90
sp|P31151|S10A7_HUMAN      ------------------------------------------------------------      101
sp|Q86SG5|S1A7A_HUMAN      ------------------------------------------------------------      101
sp|Q5SY68|S1A7B_HUMAN      ------------------------------------------------------------      101
sp|P05109|S10A8_HUMAN      ------------------------------------------------------------      93
sp|P06702|S10A9_HUMAN      ------------------------------------------------------------      114
sp|P60903|S10AA_HUMAN      ------------------------------------------------------------      97
sp|P31949|S10AB_HUMAN      ------------------------------------------------------------      105
sp|P80511|S10AC_HUMAN      ------------------------------------------------------------      92
sp|Q99584|S10AD_HUMAN      ------------------------------------------------------------      98
sp|Q9HCY8|S10AE_HUMAN      ------------------------------------------------------------      104
sp|Q96FQ6|S10AG_HUMAN      ------------------------------------------------------------      103
sp|P04271|S100B_HUMAN      ------------------------------------------------------------      92
sp|P29377|S100G_HUMAN      ------------------------------------------------------------      79
sp|P25815|S100P_HUMAN      ------------------------------------------------------------      95
sp|Q8WXG8|S100Z_HUMAN      ------------------------------------------------------------      99
sp|Q5QJ38|TCHL1_HUMAN      ------------------------------------------------------------      904
```

```
sp|Q86YZ3|HORN_HUMAN        GSRQSSGHGRQGSGSGHSPSRGRHGSGLGHSSSHGQHGSGSGRSSSRGPYESRSGHSSVF        1284

sp|P23297|S10A1_HUMAN       ------------------------------------------------------------        94
sp|P29034|S10A2_HUMAN       ------------------------------------------------------------        98
sp|P33764|S10A3_HUMAN       ------------------------------------------------------------        101
sp|P26447|S10A4_HUMAN       ------------------------------------------------------------        101
sp|P33763|S10A5_HUMAN       ------------------------------------------------------------        92
sp|P06703|S10A6_HUMAN       ------------------------------------------------------------        90
sp|P31151|S10A7_HUMAN       ------------------------------------------------------------        101
sp|Q86SG5|S1A7A_HUMAN       ------------------------------------------------------------        101
sp|Q5SY68|S1A7B_HUMAN       ------------------------------------------------------------        101
sp|P05109|S10A8_HUMAN       ------------------------------------------------------------        93
sp|P06702|S10A9_HUMAN       ------------------------------------------------------------        114
sp|P60903|S10AA_HUMAN       ------------------------------------------------------------        97
sp|P31949|S10AB_HUMAN       ------------------------------------------------------------        105
sp|P80511|S10AC_HUMAN       ------------------------------------------------------------        92
sp|Q99584|S10AD_HUMAN       ------------------------------------------------------------        98
sp|Q9HCY8|S10AE_HUMAN       ------------------------------------------------------------        104
sp|Q96FQ6|S10AG_HUMAN       ------------------------------------------------------------        103
sp|P04271|S100B_HUMAN       ------------------------------------------------------------        92
sp|P29377|S100G_HUMAN       ------------------------------------------------------------        79
sp|P25815|S100P_HUMAN       ------------------------------------------------------------        95
sp|Q8WXG8|S100Z_HUMAN       ------------------------------------------------------------        99
sp|Q5QJ38|TCHL1_HUMAN       ------------------------------------------------------------        904
sp|Q86YZ3|HORN HUMAN        GQHESGSGHSSAYSQHGSGSGHFCSQGQHGSTSGQSSTFDQEGSSTGQSSSYGHRGSGSS        1344

sp|P23297|S10A1_HUMAN       ------------------------------------------------------------        94
sp|P29034|S10A2_HUMAN       ------------------------------------------------------------        98
sp|P33764|S10A3_HUMAN       ------------------------------------------------------------        101
sp|P26447|S10A4_HUMAN       ------------------------------------------------------------        101
sp|P33763|S10A5_HUMAN       ------------------------------------------------------------        92
sp|P06703|S10A6_HUMAN       ------------------------------------------------------------        90
sp|P31151|S10A7_HUMAN       ------------------------------------------------------------        101
sp|Q86SG5|S1A7A_HUMAN       ------------------------------------------------------------        101
sp|Q5SY68|S1A7B_HUMAN       ------------------------------------------------------------        101
sp|P05109|S10A8_HUMAN       ------------------------------------------------------------        93
sp|P06702|S10A9_HUMAN       ------------------------------------------------------------        114
sp|P60903|S10AA_HUMAN       ------------------------------------------------------------        97
sp|P31949|S10AB_HUMAN       ------------------------------------------------------------        105
sp|P80511|S10AC_HUMAN       ------------------------------------------------------------        92
sp|Q99584|S10AD_HUMAN       ------------------------------------------------------------        98
sp|Q9HCY8|S10AE_HUMAN       ------------------------------------------------------------        104
sp|Q96FQ6|S10AG_HUMAN       ------------------------------------------------------------        103
sp|P04271|S100B_HUMAN       ------------------------------------------------------------        92
sp|P29377|S100G_HUMAN       ------------------------------------------------------------        79
sp|P25815|S100P_HUMAN       ------------------------------------------------------------        95
sp|Q8WXG8|S100Z_HUMAN       ------------------------------------------------------------        99
sp|Q5QJ38|TCHL1_HUMAN       ------------------------------------------------------------        904
sp|Q86YZ3|HORN_HUMAN        QSSGYGRHGAGSGQSPSRGRHGSGSGHSSSYGQHGSGSGWSSSSGRHGSGSGQSSGFGHH        1404

sp|P23297|S10A1_HUMAN       ------------------------------------------------------------        94
sp|P29034|S10A2_HUMAN       ------------------------------------------------------------        98
sp|P33764|S10A3_HUMAN       ------------------------------------------------------------        101
sp|P26447|S10A4_HUMAN       ------------------------------------------------------------        101
sp|P33763|S10A5_HUMAN       ------------------------------------------------------------        92
sp|P06703|S10A6_HUMAN       ------------------------------------------------------------        90
sp|P31151|S10A7_HUMAN       ------------------------------------------------------------        101
sp|Q86SG5|S1A7A_HUMAN       ------------------------------------------------------------        101
sp|Q5SY68|S1A7B_HUMAN       ------------------------------------------------------------        101
sp|P05109|S10A8_HUMAN       ------------------------------------------------------------        93
sp|P06702|S10A9_HUMAN       ------------------------------------------------------------        114
sp|P60903|S10AA_HUMAN       ------------------------------------------------------------        97
sp|P31949|S10AB_HUMAN       ------------------------------------------------------------        105
sp|P80511|S10AC_HUMAN       ------------------------------------------------------------        92
```

```
sp|Q99584|S10AD_HUMAN      ------------------------------------------------------------      98
sp|Q9HCY8|S10AE_HUMAN      ------------------------------------------------------------      104
sp|Q96FQ6|S10AG_HUMAN      ------------------------------------------------------------      103
sp|P04271|S100B_HUMAN      ------------------------------------------------------------      92
sp|P29377|S100G_HUMAN      ------------------------------------------------------------      79
sp|P25815|S100P_HUMAN      ------------------------------------------------------------      95
sp|Q8WXG8|S100Z_HUMAN      ------------------------------------------------------------      99
sp|Q5QJ38|TCHL1_HUMAN      ------------------------------------------------------------      904
sp|Q86YZ3|HORN_HUMAN       ESSSWQSSGCTQHGSGSGHSSSYEQHGSRSGQSSRGERHGSSSGSSSSYGQHGSGSRQSL      1464


sp|P23297|S10A1_HUMAN      ------------------------------------------------------------      94
sp|P29034|S10A2_HUMAN      ------------------------------------------------------------      98
sp|P33764|S10A3_HUMAN      ------------------------------------------------------------      101
sp|P26447|S10A4_HUMAN      ------------------------------------------------------------      101
sp|P33763|S10A5_HUMAN      ------------------------------------------------------------      92
sp|P06703|S10A6_HUMAN      ------------------------------------------------------------      90
sp|P31151|S10A7_HUMAN      ------------------------------------------------------------      101
sp|Q86SG5|S1A7A_HUMAN      ------------------------------------------------------------      101
sp|Q5SY68|S1A7B_HUMAN      ------------------------------------------------------------      101
sp|P05109|S10A8_HUMAN      ------------------------------------------------------------      93
sp|P06702|S10A9_HUMAN      ------------------------------------------------------------      114
sp|P60903|S10AA_HUMAN      ------------------------------------------------------------      97
sp|P31949|S10AB_HUMAN      ------------------------------------------------------------      105
sp|P80511|S10AC_HUMAN      ------------------------------------------------------------      92
sp|Q99584|S10AD_HUMAN      ------------------------------------------------------------      98
sp|Q9HCY8|S10AE_HUMAN      ------------------------------------------------------------      104
sp|Q96FQ6|S10AG_HUMAN      ------------------------------------------------------------      103
sp|P04271|S100B_HUMAN      ------------------------------------------------------------      92
sp|P29377|S100G_HUMAN      ------------------------------------------------------------      79
sp|P25815|S100P_HUMAN      ------------------------------------------------------------      95
sp|Q8WXG8|S100Z_HUMAN      ------------------------------------------------------------      99
sp|Q5QJ38|TCHL1_HUMAN      ------------------------------------------------------------      904
sp|Q86YZ3|HORN_HUMAN       GHGQHGSGSGQSPSPSRGRHGSGSGQSSSYSPYGSGSGWSSSRGPYESGSSHSSGLGHRE      1524


sp|P23297|S10A1_HUMAN      ------------------------------------------------------------      94
sp|P29034|S10A2_HUMAN      ------------------------------------------------------------      98
sp|P33764|S10A3_HUMAN      ------------------------------------------------------------      101
sp|P26447|S10A4_HUMAN      ------------------------------------------------------------      101
sp|P33763|S10A5_HUMAN      ------------------------------------------------------------      92
sp|P06703|S10A6_HUMAN      ------------------------------------------------------------      90
sp|P31151|S10A7_HUMAN      ------------------------------------------------------------      101
sp|Q86SG5|S1A7A_HUMAN      ------------------------------------------------------------      101
sp|Q5SY68|S1A7B_HUMAN      ------------------------------------------------------------      101
sp|P05109|S10A8_HUMAN      ------------------------------------------------------------      93
sp|P06702|S10A9_HUMAN      ------------------------------------------------------------      114
sp|P60903|S10AA_HUMAN      ------------------------------------------------------------      97
sp|P31949|S10AB_HUMAN      ------------------------------------------------------------      105
sp|P80511|S10AC_HUMAN      ------------------------------------------------------------      92
sp|Q99584|S10AD_HUMAN      ------------------------------------------------------------      98
sp|Q9HCY8|S10AE_HUMAN      ------------------------------------------------------------      104
sp|Q96FQ6|S10AG_HUMAN      ------------------------------------------------------------      103
sp|P04271|S100B_HUMAN      ------------------------------------------------------------      92
sp|P29377|S100G_HUMAN      ------------------------------------------------------------      79
sp|P25815|S100P_HUMAN      ------------------------------------------------------------      95
sp|Q8WXG8|S100Z_HUMAN      ------------------------------------------------------------      99
sp|Q5QJ38|TCHL1_HUMAN      ------------------------------------------------------------      904
sp|Q86YZ3|HORN_HUMAN       SRSGQSSGYGQHGSSSGHSSTHGQHGSTSGQSSSCGQHGASSGQSSSHGQHGSGSSQSSG      1584


sp|P23297|S10A1_HUMAN      ------------------------------------------------------------      94
sp|P29034|S10A2_HUMAN      ------------------------------------------------------------      98
sp|P33764|S10A3_HUMAN      ------------------------------------------------------------      101
sp|P26447|S10A4_HUMAN      ------------------------------------------------------------      101
sp|P33763|S10A5_HUMAN      ------------------------------------------------------------      92
sp|P06703|S10A6_HUMAN      ------------------------------------------------------------      90
```

```
sp|P31151|S10A7_HUMAN      ------------------------------------------------------------      101
sp|Q86SG5|S1A7A_HUMAN      ------------------------------------------------------------      101
sp|Q5SY68|S1A7B_HUMAN      ------------------------------------------------------------      101
sp|P05109|S10A8_HUMAN      ------------------------------------------------------------      93
sp|P06702|S10A9_HUMAN      ------------------------------------------------------------      114
sp|P60903|S10AA_HUMAN      ------------------------------------------------------------      97
sp|P31949|S10AB_HUMAN      ------------------------------------------------------------      105
sp|P80511|S10AC_HUMAN      ------------------------------------------------------------      92
sp|Q99584|S10AD_HUMAN      ------------------------------------------------------------      98
sp|Q9HCY8|S10AE_HUMAN      ------------------------------------------------------------      104
sp|Q96FQ6|S10AG_HUMAN      ------------------------------------------------------------      103
sp|P04271|S100B_HUMAN      ------------------------------------------------------------      92
sp|P29377|S100G_HUMAN      ------------------------------------------------------------      79
sp|P25815|S100P_HUMAN      ------------------------------------------------------------      95
sp|Q8WXG8|S100Z_HUMAN      ------------------------------------------------------------      99
sp|Q5QJ38|TCHL1_HUMAN      ------------------------------------------------------------      904
sp|Q86YZ3|HORN_HUMAN       YGRQGSGSGQSPGHGQRGSGSRQSPSYGRHGSGSGRSSSSGQHGSGLGESSGFGHHESSS      1644

sp|P23297|S10A1_HUMAN      ------------------------------------------------------------      94
sp|P29034|S10A2_HUMAN      ------------------------------------------------------------      98
sp|P33764|S10A3_HUMAN      ------------------------------------------------------------      101
sp|P26447|S10A4_HUMAN      ------------------------------------------------------------      101
sp|P33763|S10A5_HUMAN      ------------------------------------------------------------      92
sp|P06703|S10A6_HUMAN      ------------------------------------------------------------      90
sp|P31151|S10A7_HUMAN      ------------------------------------------------------------      101
sp|Q86SG5|S1A7A_HUMAN      ------------------------------------------------------------      101
sp|Q5SY68|S1A7B_HUMAN      ------------------------------------------------------------      101
sp|P05109|S10A8_HUMAN      ------------------------------------------------------------      93
sp|P06702|S10A9_HUMAN      ------------------------------------------------------------      114
sp|P60903|S10AA_HUMAN      ------------------------------------------------------------      97
sp|P31949|S10AB_HUMAN      ------------------------------------------------------------      105
sp|P80511|S10AC_HUMAN      ------------------------------------------------------------      92
sp|Q99584|S10AD_HUMAN      ------------------------------------------------------------      98
sp|Q9HCY8|S10AE_HUMAN      ------------------------------------------------------------      104
sp|Q96FQ6|S10AG_HUMAN      ------------------------------------------------------------      103
sp|P04271|S100B_HUMAN      ------------------------------------------------------------      92
sp|P29377|S100G_HUMAN      ------------------------------------------------------------      79
sp|P25815|S100P_HUMAN      ------------------------------------------------------------      95
sp|Q8WXG8|S100Z_HUMAN      ------------------------------------------------------------      99
sp|Q5QJ38|TCHL1_HUMAN      ------------------------------------------------------------      904
sp|Q86YZ3|HORN_HUMAN       GQSSSYSQHGSGSGHSSGYGQHGSRSGQSSRGERHGSSSRSSSRYGQHGSGSRQSSGHGR      1704

sp|P23297|S10A1_HUMAN      ------------------------------------------------------------      94
sp|P29034|S10A2_HUMAN      ------------------------------------------------------------      98
sp|P33764|S10A3_HUMAN      ------------------------------------------------------------      101
sp|P26447|S10A4_HUMAN      ------------------------------------------------------------      101
sp|P33763|S10A5_HUMAN      ------------------------------------------------------------      92
sp|P06703|S10A6_HUMAN      ------------------------------------------------------------      90
sp|P31151|S10A7_HUMAN      ------------------------------------------------------------      101
sp|Q86SG5|S1A7A_HUMAN      ------------------------------------------------------------      101
sp|Q5SY68|S1A7B_HUMAN      ------------------------------------------------------------      101
sp|P05109|S10A8_HUMAN      ------------------------------------------------------------      93
sp|P06702|S10A9_HUMAN      ------------------------------------------------------------      114
sp|P60903|S10AA_HUMAN      ------------------------------------------------------------      97
sp|P31949|S10AB_HUMAN      ------------------------------------------------------------      105
sp|P80511|S10AC_HUMAN      ------------------------------------------------------------      92
sp|Q99584|S10AD_HUMAN      ------------------------------------------------------------      98
sp|Q9HCY8|S10AE_HUMAN      ------------------------------------------------------------      104
sp|Q96FQ6|S10AG_HUMAN      ------------------------------------------------------------      103
sp|P04271|S100B_HUMAN      ------------------------------------------------------------      92
sp|P29377|S100G_HUMAN      ------------------------------------------------------------      79
sp|P25815|S100P_HUMAN      ------------------------------------------------------------      95
sp|Q8WXG8|S100Z_HUMAN      ------------------------------------------------------------      99
sp|Q5QJ38|TCHL1_HUMAN      ------------------------------------------------------------      904
sp|Q86YZ3|HORN_HUMAN       QGSGSGQSPSRGRHGSGLGHSSSHGQHGSGSGRSSSRGPYESRSGHSSVFGQHESGSGHS      1764
```

```
sp|P23297|S10A1_HUMAN      ------------------------------------------------------------      94
sp|P29034|S10A2_HUMAN      ------------------------------------------------------------      98
sp|P33764|S10A3_HUMAN      ------------------------------------------------------------      101
sp|P26447|S10A4_HUMAN      ------------------------------------------------------------      101
sp|P33763|S10A5_HUMAN      ------------------------------------------------------------      92
sp|P06703|S10A6_HUMAN      ------------------------------------------------------------      90
sp|P31151|S10A7_HUMAN      ------------------------------------------------------------      101
sp|Q86SG5|S1A7A_HUMAN      ------------------------------------------------------------      101
sp|Q5SY68|S1A7B_HUMAN      ------------------------------------------------------------      101
sp|P05109|S10A8_HUMAN      ------------------------------------------------------------      93
sp|P06702|S10A9_HUMAN      ------------------------------------------------------------      114
sp|P60903|S10AA_HUMAN      ------------------------------------------------------------      97
sp|P31949|S10AB_HUMAN      ------------------------------------------------------------      105
sp|P80511|S10AC_HUMAN      ------------------------------------------------------------      92
sp|Q99584|S10AD_HUMAN      ------------------------------------------------------------      98
sp|Q9HCY8|S10AE_HUMAN      ------------------------------------------------------------      104
sp|Q96FQ6|S10AG_HUMAN      ------------------------------------------------------------      103
sp|P04271|S100B_HUMAN      ------------------------------------------------------------      92
sp|P29377|S100G_HUMAN      ------------------------------------------------------------      79
sp|P25815|S100P_HUMAN      ------------------------------------------------------------      95
sp|Q8WXG8|S100Z_HUMAN      ------------------------------------------------------------      99
sp|Q5QJ38|TCHL1_HUMAN      ------------------------------------------------------------      904
sp|Q86YZ3|HORN_HUMAN       SAYSQHGSGSGHFCSQGQHGSTSGQSSTFDQEGSSTGQSSSHGQHGSGSSQSSSYGQQGS      1824

sp|P23297|S10A1_HUMAN      ------------------------------------------------------------      94
sp|P29034|S10A2_HUMAN      ------------------------------------------------------------      98
sp|P33764|S10A3_HUMAN      ------------------------------------------------------------      101
sp|P26447|S10A4_HUMAN      ------------------------------------------------------------      101
sp|P33763|S10A5_HUMAN      ------------------------------------------------------------      92
sp|P06703|S10A6_HUMAN      ------------------------------------------------------------      90
sp|P31151|S10A7_HUMAN      ------------------------------------------------------------      101
sp|Q86SG5|S1A7A_HUMAN      ------------------------------------------------------------      101
sp|Q5SY68|S1A7B_HUMAN      ------------------------------------------------------------      101
sp|P05109|S10A8_HUMAN      ------------------------------------------------------------      93
sp|P06702|S10A9_HUMAN      ------------------------------------------------------------      114
sp|P60903|S10AA_HUMAN      ------------------------------------------------------------      97
sp|P31949|S10AB_HUMAN      ------------------------------------------------------------      105
sp|P80511|S10AC_HUMAN      ------------------------------------------------------------      92
sp|Q99584|S10AD_HUMAN      ------------------------------------------------------------      98
sp|Q9HCY8|S10AE_HUMAN      ------------------------------------------------------------      104
sp|Q96FQ6|S10AG_HUMAN      ------------------------------------------------------------      103
sp|P04271|S100B_HUMAN      ------------------------------------------------------------      92
sp|P29377|S100G_HUMAN      ------------------------------------------------------------      79
sp|P25815|S100P_HUMAN      ------------------------------------------------------------      95
sp|Q8WXG8|S100Z_HUMAN      ------------------------------------------------------------      99
sp|Q5QJ38|TCHL1_HUMAN      ------------------------------------------------------------      904
sp|Q86YZ3|HORN_HUMAN       GSGQSPSRGRHGSGSGHSSSYGQHGSGSGWSSSSGRHGSGSGQSSGFGHHESSSWQSSGY      1884

sp|P23297|S10A1_HUMAN      ------------------------------------------------------------      94
sp|P29034|S10A2_HUMAN      ------------------------------------------------------------      98
sp|P33764|S10A3_HUMAN      ------------------------------------------------------------      101
sp|P26447|S10A4_HUMAN      ------------------------------------------------------------      101
sp|P33763|S10A5_HUMAN      ------------------------------------------------------------      92
sp|P06703|S10A6_HUMAN      ------------------------------------------------------------      90
sp|P31151|S10A7_HUMAN      ------------------------------------------------------------      101
sp|Q86SG5|S1A7A_HUMAN      ------------------------------------------------------------      101
sp|Q5SY68|S1A7B_HUMAN      ------------------------------------------------------------      101
sp|P05109|S10A8_HUMAN      ------------------------------------------------------------      93
sp|P06702|S10A9_HUMAN      ------------------------------------------------------------      114
sp|P60903|S10AA_HUMAN      ------------------------------------------------------------      97
sp|P31949|S10AB_HUMAN      ------------------------------------------------------------      105
sp|P80511|S10AC_HUMAN      ------------------------------------------------------------      92
sp|Q99584|S10AD_HUMAN      ------------------------------------------------------------      98
```

```
sp|Q9HCY8|S10AE_HUMAN      ------------------------------------------------------------      104
sp|Q96FQ6|S10AG_HUMAN      ------------------------------------------------------------      103
sp|P04271|S100B_HUMAN      ------------------------------------------------------------      92
sp|P29377|S100G_HUMAN      ------------------------------------------------------------      79
sp|P25815|S100P_HUMAN      ------------------------------------------------------------      95
sp|Q8WXG8|S100Z_HUMAN      ------------------------------------------------------------      99
sp|Q5QJ38|TCHL1_HUMAN      ------------------------------------------------------------      904
sp|Q86YZ3|HORN_HUMAN       TQHGSGSGHSSSYEQHGSRSGQSSRGEQHGSSSGSSSSYGQHGSGSRQSLGHGQHGSGSG      1944

sp|P23297|S10A1_HUMAN      ------------------------------------------------------------      94
sp|P29034|S10A2_HUMAN      ------------------------------------------------------------      98
sp|P33764|S10A3_HUMAN      ------------------------------------------------------------      101
sp|P26447|S10A4_HUMAN      ------------------------------------------------------------      101
sp|P33763|S10A5_HUMAN      ------------------------------------------------------------      92
sp|P06703|S10A6_HUMAN      ------------------------------------------------------------      90
sp|P31151|S10A7_HUMAN      ------------------------------------------------------------      101
sp|Q86SG5|S1A7A_HUMAN      ------------------------------------------------------------      101
sp|Q5SY68|S1A7B_HUMAN      ------------------------------------------------------------      101
sp|P05109|S10A8_HUMAN      ------------------------------------------------------------      93
sp|P06702|S10A9_HUMAN      ------------------------------------------------------------      114
sp|P60903|S10AA_HUMAN      ------------------------------------------------------------      97
sp|P31949|S10AB_HUMAN      ------------------------------------------------------------      105
sp|P80511|S10AC_HUMAN      ------------------------------------------------------------      92
sp|Q99584|S10AD_HUMAN      ------------------------------------------------------------      98
sp|Q9HCY8|S10AE_HUMAN      ------------------------------------------------------------      104
sp|Q96FQ6|S10AG_HUMAN      ------------------------------------------------------------      103
sp|P04271|S100B_HUMAN      ------------------------------------------------------------      92
sp|P29377|S100G_HUMAN      ------------------------------------------------------------      79
sp|P25815|S100P_HUMAN      ------------------------------------------------------------      95
sp|Q8WXG8|S100Z_HUMAN      ------------------------------------------------------------      99
sp|Q5QJ38|TCHL1_HUMAN      ------------------------------------------------------------      904
sp|Q86YZ3|HORN_HUMAN       QSPSPSRGRHGSGSGQSSSYGPYGSGSGWSSSRGPYESGSGHSSGLGHRESRSGQSSGYG      2004

sp|P23297|S10A1_HUMAN      ------------------------------------------------------------      94
sp|P29034|S10A2_HUMAN      ------------------------------------------------------------      98
sp|P33764|S10A3_HUMAN      ------------------------------------------------------------      101
sp|P26447|S10A4_HUMAN      ------------------------------------------------------------      101
sp|P33763|S10A5_HUMAN      ------------------------------------------------------------      92
sp|P06703|S10A6_HUMAN      ------------------------------------------------------------      90
sp|P31151|S10A7_HUMAN      ------------------------------------------------------------      101
sp|Q86SG5|S1A7A_HUMAN      ------------------------------------------------------------      101
sp|Q5SY68|S1A7B_HUMAN      ------------------------------------------------------------      101
sp|P05109|S10A8_HUMAN      ------------------------------------------------------------      93
sp|P06702|S10A9_HUMAN      ------------------------------------------------------------      114
sp|P60903|S10AA_HUMAN      ------------------------------------------------------------      97
sp|P31949|S10AB_HUMAN      ------------------------------------------------------------      105
sp|P80511|S10AC_HUMAN      ------------------------------------------------------------      92
sp|Q99584|S10AD_HUMAN      ------------------------------------------------------------      98
sp|Q9HCY8|S10AE_HUMAN      ------------------------------------------------------------      104
sp|Q96FQ6|S10AG_HUMAN      ------------------------------------------------------------      103
sp|P04271|S100B_HUMAN      ------------------------------------------------------------      92
sp|P29377|S100G_HUMAN      ------------------------------------------------------------      79
sp|P25815|S100P_HUMAN      ------------------------------------------------------------      95
sp|Q8WXG8|S100Z_HUMAN      ------------------------------------------------------------      99
sp|Q5QJ38|TCHL1_HUMAN      ------------------------------------------------------------      904
sp|Q86YZ3|HORN_HUMAN       QHGSSSGHSSTHGQHGSASGQSSSCGQHGASSGQSSSHGQHGSGSSQSSGYGRQGSGSGQ      2064

sp|P23297|S10A1_HUMAN      ------------------------------------------------------------      94
sp|P29034|S10A2_HUMAN      ------------------------------------------------------------      98
sp|P33764|S10A3_HUMAN      ------------------------------------------------------------      101
sp|P26447|S10A4_HUMAN      ------------------------------------------------------------      101
sp|P33763|S10A5_HUMAN      ------------------------------------------------------------      92
sp|P06703|S10A6_HUMAN      ------------------------------------------------------------      90
sp|P31151|S10A7_HUMAN      ------------------------------------------------------------      101
```

```
sp|Q86SG5|S1A7A_HUMAN      ------------------------------------------------------------      101
sp|Q5SY68|S1A7B_HUMAN      ------------------------------------------------------------      101
sp|P05109|S10A8_HUMAN      ------------------------------------------------------------      93
sp|P06702|S10A9_HUMAN      ------------------------------------------------------------      114
sp|P60903|S10AA_HUMAN      ------------------------------------------------------------      97
sp|P31949|S10AB_HUMAN      ------------------------------------------------------------      105
sp|P80511|S10AC_HUMAN      ------------------------------------------------------------      92
sp|Q99584|S10AD_HUMAN      ------------------------------------------------------------      98
sp|Q9HCY8|S10AE_HUMAN      ------------------------------------------------------------      104
sp|Q96FQ6|S10AG_HUMAN      ------------------------------------------------------------      103
sp|P04271|S100B_HUMAN      ------------------------------------------------------------      92
sp|P29377|S100G_HUMAN      ------------------------------------------------------------      79
sp|P25815|S100P_HUMAN      ------------------------------------------------------------      95
sp|Q8WXG8|S100Z_HUMAN      ------------------------------------------------------------      99
sp|Q5QJ38|TCHL1_HUMAN      ------------------------------------------------------------      904
sp|Q86YZ3|HORN_HUMAN       SPGHGQRGSGSRQSPSYGRHGSGSGRSSSSGQHGPGLGESSGFGHHESSSGQSSSYSQHG      2124

sp|P23297|S10A1_HUMAN      ------------------------------------------------------------      94
sp|P29034|S10A2_HUMAN      ------------------------------------------------------------      98
sp|P33764|S10A3_HUMAN      ------------------------------------------------------------      101
sp|P26447|S10A4_HUMAN      ------------------------------------------------------------      101
sp|P33763|S10A5_HUMAN      ------------------------------------------------------------      92
sp|P06703|S10A6_HUMAN      ------------------------------------------------------------      90
sp|P31151|S10A7_HUMAN      ------------------------------------------------------------      101
sp|Q86SG5|S1A7A_HUMAN      ------------------------------------------------------------      101
sp|Q5SY68|S1A7B_HUMAN      ------------------------------------------------------------      101
sp|P05109|S10A8_HUMAN      ------------------------------------------------------------      93
sp|P06702|S10A9_HUMAN      ------------------------------------------------------------      114
sp|P60903|S10AA_HUMAN      ------------------------------------------------------------      97
sp|P31949|S10AB_HUMAN      ------------------------------------------------------------      105
sp|P80511|S10AC_HUMAN      ------------------------------------------------------------      92
sp|Q99584|S10AD_HUMAN      ------------------------------------------------------------      98
sp|Q9HCY8|S10AE_HUMAN      ------------------------------------------------------------      104
sp|Q96FQ6|S10AG_HUMAN      ------------------------------------------------------------      103
sp|P04271|S100B_HUMAN      ------------------------------------------------------------      92
sp|P29377|S100G_HUMAN      ------------------------------------------------------------      79
sp|P25815|S100P_HUMAN      ------------------------------------------------------------      95
sp|Q8WXG8|S100Z_HUMAN      ------------------------------------------------------------      99
sp|Q5QJ38|TCHL1_HUMAN      ------------------------------------------------------------      904
sp|Q86YZ3|HORN_HUMAN       SGSGHSSGYGQHGSRSGQSSRGERHGSSSGSSSRYGQHGSGSRQSSGHGRQGSGSGHSPS      2184

sp|P23297|S10A1_HUMAN      ------------------------------------------------------------      94
sp|P29034|S10A2_HUMAN      ------------------------------------------------------------      98
sp|P33764|S10A3_HUMAN      ------------------------------------------------------------      101
sp|P26447|S10A4_HUMAN      ------------------------------------------------------------      101
sp|P33763|S10A5_HUMAN      ------------------------------------------------------------      92
sp|P06703|S10A6_HUMAN      ------------------------------------------------------------      90
sp|P31151|S10A7_HUMAN      ------------------------------------------------------------      101
sp|Q86SG5|S1A7A_HUMAN      ------------------------------------------------------------      101
sp|Q5SY68|S1A7B_HUMAN      ------------------------------------------------------------      101
sp|P05109|S10A8_HUMAN      ------------------------------------------------------------      93
sp|P06702|S10A9_HUMAN      ------------------------------------------------------------      114
sp|P60903|S10AA_HUMAN      ------------------------------------------------------------      97
sp|P31949|S10AB_HUMAN      ------------------------------------------------------------      105
sp|P80511|S10AC_HUMAN      ------------------------------------------------------------      92
sp|Q99584|S10AD_HUMAN      ------------------------------------------------------------      98
sp|Q9HCY8|S10AE_HUMAN      ------------------------------------------------------------      104
sp|Q96FQ6|S10AG_HUMAN      ------------------------------------------------------------      103
sp|P04271|S100B_HUMAN      ------------------------------------------------------------      92
sp|P29377|S100G_HUMAN      ------------------------------------------------------------      79
sp|P25815|S100P_HUMAN      ------------------------------------------------------------      95
sp|Q8WXG8|S100Z_HUMAN      ------------------------------------------------------------      99
sp|Q5QJ38|TCHL1_HUMAN      ------------------------------------------------------------      904
sp|Q86YZ3|HORN_HUMAN       RGRHGSGSGHSSSHGQHGSGSGRSSSRGPYESRSGHSSVFGQHESGSGHSSAYSQHGSGS      2244
```

```
sp|P23297|S10A1_HUMAN      ------------------------------------------------------------      94
sp|P29034|S10A2_HUMAN      ------------------------------------------------------------      98
sp|P33764|S10A3_HUMAN      ------------------------------------------------------------      101
sp|P26447|S10A4_HUMAN      ------------------------------------------------------------      101
sp|P33763|S10A5_HUMAN      ------------------------------------------------------------      92
sp|P06703|S10A6_HUMAN      ------------------------------------------------------------      90
sp|P31151|S10A7_HUMAN      ------------------------------------------------------------      101
sp|Q86SG5|S1A7A_HUMAN      ------------------------------------------------------------      101
sp|Q5SY68|S1A7B_HUMAN      ------------------------------------------------------------      101
sp|P05109|S10A8_HUMAN      ------------------------------------------------------------      93
sp|P06702|S10A9_HUMAN      ------------------------------------------------------------      114
sp|P60903|S10AA_HUMAN      ------------------------------------------------------------      97
sp|P31949|S10AB_HUMAN      ------------------------------------------------------------      105
sp|P80511|S10AC_HUMAN      ------------------------------------------------------------      92
sp|Q99584|S10AD_HUMAN      ------------------------------------------------------------      98
sp|Q9HCY8|S10AE_HUMAN      ------------------------------------------------------------      104
sp|Q96FQ6|S10AG_HUMAN      ------------------------------------------------------------      103
sp|P04271|S100B_HUMAN      ------------------------------------------------------------      92
sp|P29377|S100G_HUMAN      ------------------------------------------------------------      79
sp|P25815|S100P_HUMAN      ------------------------------------------------------------      95
sp|Q8WXG8|S100Z_HUMAN      ------------------------------------------------------------      99
sp|Q5QJ38|TCHL1_HUMAN      ------------------------------------------------------------      904
sp|Q86YZ3|HORN_HUMAN       GHFCSQGQHGSTSGQSSTFDQEGSSTGQSSSHGQHGSGSSQSSSYGQQGSGSGQSPSRGR      2304

sp|P23297|S10A1_HUMAN      ------------------------------------------------------------      94
sp|P29034|S10A2_HUMAN      ------------------------------------------------------------      98
sp|P33764|S10A3_HUMAN      ------------------------------------------------------------      101
sp|P26447|S10A4_HUMAN      ------------------------------------------------------------      101
sp|P33763|S10A5_HUMAN      ------------------------------------------------------------      92
sp|P06703|S10A6_HUMAN      ------------------------------------------------------------      90
sp|P31151|S10A7_HUMAN      ------------------------------------------------------------      101
sp|Q86SG5|S1A7A_HUMAN      ------------------------------------------------------------      101
sp|Q5SY68|S1A7B_HUMAN      ------------------------------------------------------------      101
sp|P05109|S10A8_HUMAN      ------------------------------------------------------------      93
sp|P06702|S10A9_HUMAN      ------------------------------------------------------------      114
sp|P60903|S10AA_HUMAN      ------------------------------------------------------------      97
sp|P31949|S10AB_HUMAN      ------------------------------------------------------------      105
sp|P80511|S10AC_HUMAN      ------------------------------------------------------------      92
sp|Q99584|S10AD_HUMAN      ------------------------------------------------------------      98
sp|Q9HCY8|S10AE_HUMAN      ------------------------------------------------------------      104
sp|Q96FQ6|S10AG_HUMAN      ------------------------------------------------------------      103
sp|P04271|S100B_HUMAN      ------------------------------------------------------------      92
sp|P29377|S100G_HUMAN      ------------------------------------------------------------      79
sp|P25815|S100P_HUMAN      ------------------------------------------------------------      95
sp|Q8WXG8|S100Z_HUMAN      ------------------------------------------------------------      99
sp|Q5QJ38|TCHL1_HUMAN      ------------------------------------------------------------      904
sp|Q86YZ3|HORN_HUMAN       HGSGSGHSSSYGQHGSGSGWSSSSGRHGSGSGQSSGFGHHESSSWQSSGYTQHGSGSGHS      2364

sp|P23297|S10A1 HUMAN      ------------------------------------------------------------      94
sp|P29034|S10A2_HUMAN      ------------------------------------------------------------      98
sp|P33764|S10A3_HUMAN      ------------------------------------------------------------      101
sp|P26447|S10A4_HUMAN      ------------------------------------------------------------      101
sp|P33763|S10A5_HUMAN      ------------------------------------------------------------      92
sp|P06703|S10A6_HUMAN      ------------------------------------------------------------      90
sp|P31151|S10A7_HUMAN      ------------------------------------------------------------      101
sp|Q86SG5|S1A7A_HUMAN      ------------------------------------------------------------      101
sp|Q5SY68|S1A7B_HUMAN      ------------------------------------------------------------      101
sp|P05109|S10A8_HUMAN      ------------------------------------------------------------      93
sp|P06702|S10A9_HUMAN      ------------------------------------------------------------      114
sp|P60903|S10AA_HUMAN      ------------------------------------------------------------      97
sp|P31949|S10AB_HUMAN      ------------------------------------------------------------      105
sp|P80511|S10AC_HUMAN      ------------------------------------------------------------      92
sp|Q99584|S10AD_HUMAN      ------------------------------------------------------------      98
sp|Q9HCY8|S10AE_HUMAN      ------------------------------------------------------------      104
```

```
sp|Q96FQ6|S10AG_HUMAN      ------------------------------------------------------------      103
sp|P04271|S100B_HUMAN      ------------------------------------------------------------      92
sp|P29377|S100G_HUMAN      ------------------------------------------------------------      79
sp|P25815|S100P_HUMAN      ------------------------------------------------------------      95
sp|Q8WXG8|S100Z_HUMAN      ------------------------------------------------------------      99
sp|Q5QJ38|TCHL1_HUMAN      ------------------------------------------------------------      904
sp|Q86YZ3|HORN_HUMAN       SSYEQHGSRSGQSSRGERHGSSSGSSSSYGQHGSGSRQSLGHGQHGSGSGQSPSPSRGRH      2424

sp|P23297|S10A1_HUMAN      ------------------------------------------------------------      94
sp|P29034|S10A2_HUMAN      ------------------------------------------------------------      98
sp|P33764|S10A3_HUMAN      ------------------------------------------------------------      101
sp|P26447|S10A4_HUMAN      ------------------------------------------------------------      101
sp|P33763|S10A5_HUMAN      ------------------------------------------------------------      92
sp|P06703|S10A6_HUMAN      ------------------------------------------------------------      90
sp|P31151|S10A7_HUMAN      ------------------------------------------------------------      101
sp|Q86SG5|S1A7A_HUMAN      ------------------------------------------------------------      101
sp|Q5SY68|S1A7B_HUMAN      ------------------------------------------------------------      101
sp|P05109|S10A8_HUMAN      ------------------------------------------------------------      93
sp|P06702|S10A9_HUMAN      ------------------------------------------------------------      114
sp|P60903|S10AA_HUMAN      ------------------------------------------------------------      97
sp|P31949|S10AB_HUMAN      ------------------------------------------------------------      105
sp|P80511|S10AC_HUMAN      ------------------------------------------------------------      92
sp|Q99584|S10AD_HUMAN      ------------------------------------------------------------      98
sp|Q9HCY8|S10AE_HUMAN      ------------------------------------------------------------      104
sp|Q96FQ6|S10AG_HUMAN      ------------------------------------------------------------      103
sp|P04271|S100B_HUMAN      ------------------------------------------------------------      92
sp|P29377|S100G_HUMAN      ------------------------------------------------------------      79
sp|P25815|S100P_HUMAN      ------------------------------------------------------------      95
sp|Q8WXG8|S100Z_HUMAN      ------------------------------------------------------------      99
sp|Q5QJ38|TCHL1_HUMAN      ------------------------------------------------------------      904
sp|Q86YZ3|HORN_HUMAN       GSGSGQSSSYSPYGSGSGWSSSRGPYESGSGHSSGLGHRESRSGQSSGYGQHGSSSGHSS      2484

sp|P23297|S10A1_HUMAN      ------------------------------------------------------------      94
sp|P29034|S10A2_HUMAN      ------------------------------------------------------------      98
sp|P33764|S10A3_HUMAN      ------------------------------------------------------------      101
sp|P26447|S10A4_HUMAN      ------------------------------------------------------------      101
sp|P33763|S10A5_HUMAN      ------------------------------------------------------------      92
sp|P06703|S10A6_HUMAN      ------------------------------------------------------------      90
sp|P31151|S10A7_HUMAN      ------------------------------------------------------------      101
sp|Q86SG5|S1A7A_HUMAN      ------------------------------------------------------------      101
sp|Q5SY68|S1A7B_HUMAN      ------------------------------------------------------------      101
sp|P05109|S10A8_HUMAN      ------------------------------------------------------------      93
sp|P06702|S10A9_HUMAN      ------------------------------------------------------------      114
sp|P60903|S10AA_HUMAN      ------------------------------------------------------------      97
sp|P31949|S10AB_HUMAN      ------------------------------------------------------------      105
sp|P80511|S10AC_HUMAN      ------------------------------------------------------------      92
sp|Q99584|S10AD_HUMAN      ------------------------------------------------------------      98
sp|Q9HCY8|S10AE_HUMAN      ------------------------------------------------------------      104
sp|Q96FQ6|S10AG_HUMAN      ------------------------------------------------------------      103
sp|P04271|S100B_HUMAN      ------------------------------------------------------------      92
sp|P29377|S100G_HUMAN      ------------------------------------------------------------      79
sp|P25815|S100P_HUMAN      ------------------------------------------------------------      95
sp|Q8WXG8|S100Z_HUMAN      ------------------------------------------------------------      99
sp|Q5QJ38|TCHL1_HUMAN      ------------------------------------------------------------      904
sp|Q86YZ3|HORN_HUMAN       THGQHGSTSGQSSSCGQHGASSGQSSSHGQHGSGSSQSSGYGRQGSGSGQSPGHGQRGSG      2544

sp|P23297|S10A1_HUMAN      ------------------------------------------------------------      94
sp|P29034|S10A2_HUMAN      ------------------------------------------------------------      98
sp|P33764|S10A3_HUMAN      ------------------------------------------------------------      101
sp|P26447|S10A4_HUMAN      ------------------------------------------------------------      101
sp|P33763|S10A5_HUMAN      ------------------------------------------------------------      92
sp|P06703|S10A6_HUMAN      ------------------------------------------------------------      90
sp|P31151|S10A7_HUMAN      ------------------------------------------------------------      101
sp|Q86SG5|S1A7A_HUMAN      ------------------------------------------------------------      101
```

```
sp|Q5SY68|S1A7B_HUMAN      ------------------------------------------------------------      101
sp|P05109|S10A8_HUMAN      ------------------------------------------------------------      93
sp|P06702|S10A9_HUMAN      ------------------------------------------------------------      114
sp|P60903|S10AA_HUMAN      ------------------------------------------------------------      97
sp|P31949|S10AB_HUMAN      ------------------------------------------------------------      105
sp|P80511|S10AC_HUMAN      ------------------------------------------------------------      92
sp|Q99584|S10AD_HUMAN      ------------------------------------------------------------      98
sp|Q9HCY8|S10AE_HUMAN      ------------------------------------------------------------      104
sp|Q96FQ6|S10AG_HUMAN      ------------------------------------------------------------      103
sp|P04271|S100B_HUMAN      ------------------------------------------------------------      92
sp|P29377|S100G_HUMAN      ------------------------------------------------------------      79
sp|P25815|S100P_HUMAN      ------------------------------------------------------------      95
sp|Q8WXG8|S100Z_HUMAN      ------------------------------------------------------------      99
sp|Q5QJ38|TCHL1_HUMAN      ------------------------------------------------------------      904
sp|Q86YZ3|HORN_HUMAN       SRQSPSYGRHGSGSGRSSSSGQHGSGLGESSGFGHHESSSGQSSSYSQHGSGSGHSSGYG      2604

sp|P23297|S10A1_HUMAN      ------------------------------------------------------------      94
sp|P29034|S10A2_HUMAN      ------------------------------------------------------------      98
sp|P33764|S10A3_HUMAN      ------------------------------------------------------------      101
sp|P26447|S10A4_HUMAN      ------------------------------------------------------------      101
sp|P33763|S10A5_HUMAN      ------------------------------------------------------------      92
sp|P06703|S10A6_HUMAN      ------------------------------------------------------------      90
sp|P31151|S10A7_HUMAN      ------------------------------------------------------------      101
sp|Q86SG5|S1A7A_HUMAN      ------------------------------------------------------------      101
sp|Q5SY68|S1A7B_HUMAN      ------------------------------------------------------------      101
sp|P05109|S10A8_HUMAN      ------------------------------------------------------------      93
sp|P06702|S10A9_HUMAN      ------------------------------------------------------------      114
sp|P60903|S10AA_HUMAN      ------------------------------------------------------------      97
sp|P31949|S10AB_HUMAN      ------------------------------------------------------------      105
sp|P80511|S10AC_HUMAN      ------------------------------------------------------------      92
sp|Q99584|S10AD_HUMAN      ------------------------------------------------------------      98
sp|Q9HCY8|S10AE_HUMAN      ------------------------------------------------------------      104
sp|Q96FQ6|S10AG_HUMAN      ------------------------------------------------------------      103
sp|P04271|S100B_HUMAN      ------------------------------------------------------------      92
sp|P29377|S100G_HUMAN      ------------------------------------------------------------      79
sp|P25815|S100P_HUMAN      ------------------------------------------------------------      95
sp|Q8WXG8|S100Z_HUMAN      ------------------------------------------------------------      99
sp|Q5QJ38|TCHL1_HUMAN      ------------------------------------------------------------      904
sp|Q86YZ3|HORN_HUMAN       QHGSRSGQSSRGERHGSSSGSSSHYGQHGSGSRQSSGHGRQGSGSGQSPSRGRHGSGLGH      2664

sp|P23297|S10A1_HUMAN      ------------------------------------------------------------      94
sp|P29034|S10A2_HUMAN      ------------------------------------------------------------      98
sp|P33764|S10A3_HUMAN      ------------------------------------------------------------      101
sp|P26447|S10A4_HUMAN      ------------------------------------------------------------      101
sp|P33763|S10A5_HUMAN      ------------------------------------------------------------      92
sp|P06703|S10A6_HUMAN      ------------------------------------------------------------      90
sp|P31151|S10A7_HUMAN      ------------------------------------------------------------      101
sp|Q86SG5|S1A7A_HUMAN      ------------------------------------------------------------      101
sp|Q5SY68|S1A7B_HUMAN      ------------------------------------------------------------      101
sp|P05109|S10A8_HUMAN      ------------------------------------------------------------      93
sp|P06702|S10A9_HUMAN      ------------------------------------------------------------      114
sp|P60903|S10AA_HUMAN      ------------------------------------------------------------      97
sp|P31949|S10AB_HUMAN      ------------------------------------------------------------      105
sp|P80511|S10AC_HUMAN      ------------------------------------------------------------      92
sp|Q99584|S10AD_HUMAN      ------------------------------------------------------------      98
sp|Q9HCY8|S10AE_HUMAN      ------------------------------------------------------------      104
sp|Q96FQ6|S10AG_HUMAN      ------------------------------------------------------------      103
sp|P04271|S100B_HUMAN      ------------------------------------------------------------      92
sp|P29377|S100G_HUMAN      ------------------------------------------------------------      79
sp|P25815|S100P_HUMAN      ------------------------------------------------------------      95
sp|Q8WXG8|S100Z_HUMAN      ------------------------------------------------------------      99
sp|Q5QJ38|TCHL1_HUMAN      ------------------------------------------------------------      904
sp|Q86YZ3|HORN_HUMAN       SSSHGQHGSGSGRSSSRGPYESRLGHSSVFGQHESGSGHSSAYSQHGSGSGHFCSQGQHG      2724
```

```
sp|P23297|S10A1_HUMAN      ------------------------------------------------------------        94
sp|P29034|S10A2_HUMAN      ------------------------------------------------------------        98
sp|P33764|S10A3_HUMAN      ------------------------------------------------------------        101
sp|P26447|S10A4_HUMAN      ------------------------------------------------------------        101
sp|P33763|S10A5_HUMAN      ------------------------------------------------------------        92
sp|P06703|S10A6_HUMAN      ------------------------------------------------------------        90
sp|P31151|S10A7_HUMAN      ------------------------------------------------------------        101
sp|Q86SG5|S1A7A_HUMAN      ------------------------------------------------------------        101
sp|Q5SY68|S1A7B_HUMAN      ------------------------------------------------------------        101
sp|P05109|S10A8_HUMAN      ------------------------------------------------------------        93
sp|P06702|S10A9_HUMAN      ------------------------------------------------------------        114
sp|P60903|S10AA_HUMAN      ------------------------------------------------------------        97
sp|P31949|S10AB_HUMAN      ------------------------------------------------------------        105
sp|P80511|S10AC_HUMAN      ------------------------------------------------------------        92
sp|Q99584|S10AD_HUMAN      ------------------------------------------------------------        98
sp|Q9HCY8|S10AE_HUMAN      ------------------------------------------------------------        104
sp|Q96FQ6|S10AG_HUMAN      ------------------------------------------------------------        103
sp|P04271|S100B_HUMAN      ------------------------------------------------------------        92
sp|P29377|S100G_HUMAN      ------------------------------------------------------------        79
sp|P25815|S100P_HUMAN      ------------------------------------------------------------        95
sp|Q8WXG8|S100Z_HUMAN      ------------------------------------------------------------        99
sp|Q5QJ38|TCHL1_HUMAN      ------------------------------------------------------------        904
sp|Q86YZ3|HORN_HUMAN       STSGQSSTFDQEGSSTGQSSSYGHRGSGSSQSSGYGRHGAGSGQSLSHGRHGSGSGQSSS        2784

sp|P23297|S10A1_HUMAN      ------------------------------------------------------------        94
sp|P29034|S10A2_HUMAN      ------------------------------------------------------------        98
sp|P33764|S10A3_HUMAN      ------------------------------------------------------------        101
sp|P26447|S10A4_HUMAN      ------------------------------------------------------------        101
sp|P33763|S10A5_HUMAN      ------------------------------------------------------------        92
sp|P06703|S10A6_HUMAN      ------------------------------------------------------------        90
sp|P31151|S10A7_HUMAN      ------------------------------------------------------------        101
sp|Q86SG5|S1A7A_HUMAN      ------------------------------------------------------------        101
sp|Q5SY68|S1A7B_HUMAN      ------------------------------------------------------------        101
sp|P05109|S10A8_HUMAN      ------------------------------------------------------------        93
sp|P06702|S10A9_HUMAN      ------------------------------------------------------------        114
sp|P60903|S10AA_HUMAN      ------------------------------------------------------------        97
sp|P31949|S10AB_HUMAN      ------------------------------------------------------------        105
sp|P80511|S10AC_HUMAN      ------------------------------------------------------------        92
sp|Q99584|S10AD_HUMAN      ------------------------------------------------------------        98
sp|Q9HCY8|S10AE_HUMAN      ------------------------------------------------------------        104
sp|Q96FQ6|S10AG_HUMAN      ------------------------------------------------------------        103
sp|P04271|S100B_HUMAN      ------------------------------------------------------------        92
sp|P29377|S100G_HUMAN      ------------------------------------------------------------        79
sp|P25815|S100P_HUMAN      ------------------------------------------------------------        95
sp|Q8WXG8|S100Z_HUMAN      ------------------------------------------------------------        99
sp|Q5QJ38|TCHL1_HUMAN      ------------------------------------------------------------        904
sp|Q86YZ3|HORN_HUMAN       YGQHGSGSGQSSGYSQHGSGSGQDGYSYCKGGSNHDGGSSGSYFLSFPSSTSPYEYVQEQ        2844

sp|P23297|S10A1_HUMAN      ------  94
sp|P29034|S10A2_HUMAN      ------  98
sp|P33764|S10A3_HUMAN      ------  101
sp|P26447|S10A4_HUMAN      ------  101
sp|P33763|S10A5_HUMAN      ------  92
sp|P06703|S10A6_HUMAN      ------  90
sp|P31151|S10A7_HUMAN      ------  101
sp|Q86SG5|S1A7A_HUMAN      ------  101
sp|Q5SY68|S1A7B_HUMAN      ------  101
sp|P05109|S10A8_HUMAN      ------  93
sp|P06702|S10A9_HUMAN      ------  114
sp|P60903|S10AA_HUMAN      ------  97
sp|P31949|S10AB_HUMAN      ------  105
sp|P80511|S10AC_HUMAN      ------  92
sp|Q99584|S10AD_HUMAN      ------  98
sp|Q9HCY8|S10AE_HUMAN      ------  104
sp|Q96FQ6|S10AG_HUMAN      ------  103
```

```
sp|P04271|S100B_HUMAN      ------  92
sp|P29377|S100G_HUMAN      ------  79
sp|P25815|S100P_HUMAN      ------  95
sp|Q8WXG8|S100Z_HUMAN      ------  99
sp|Q5QJ38|TCHL1_HUMAN      ------  904
sp|Q86YZ3|HORN_HUMAN       RCYFYQ  2850
```

Percent Identity Matrix - created by Clustal2.1
#
#

sp|P23297|S10A1_HUMAN	100.00	50.00	40.43	48.94	46.15	43.33	20.88	23.08	20.88	37.78	36.17	47.25	40.43	36.96	34.07	24.42	32.98	57.61	40.51	52.69	57.45	23.91	32.26
sp|P29034|S10A2_HUMAN	50.00	100.00	43.30	60.82	52.17	47.78	21.05	22.11	22.11	27.96	30.61	32.98	28.57	34.78	29.47	23.86	29.59	43.48	35.44	40.00	40.21	18.95	25.00
sp|P33764|S10A3_HUMAN	40.43	43.30	100.00	44.55	40.22	45.56	16.33	18.37	19.39	21.51	29.70	25.77	27.00	30.43	26.60	18.68	23.23	36.96	25.32	31.58	34.34	22.22	25.00
sp|P26447|S10A4_HUMAN	48.94	60.82	44.55	100.00	52.17	51.11	22.45	24.49	22.45	29.03	27.72	34.02	28.00	32.61	29.79	25.27	31.31	45.65	36.71	41.05	43.43	20.20	25.00
sp|P33763|S10A5_HUMAN	46.15	52.17	40.22	52.17	100.00	51.69	24.72	25.84	19.10	30.68	32.61	33.71	32.61	34.83	36.67	21.43	29.35	42.70	35.06	38.46	42.39	22.22	28.57
sp|P06703|S10A6_HUMAN	43.33	47.78	45.56	51.11	51.69	100.00	22.99	25.29	22.99	27.91	30.00	26.44	28.89	34.83	29.89	25.00	27.78	39.33	35.06	34.83	38.89	22.73	25.84
sp|P31151|S10A7_HUMAN	20.88	21.05	16.33	22.45	24.72	22.99	100.00	94.06	52.48	24.44	24.00	22.68	32.65	25.84	19.57	19.10	25.77	25.84	28.95	27.17	25.00	16.67	19.59
sp|Q86SG5|S1A7A_HUMAN	23.08	22.11	18.37	24.49	25.84	25.29	94.06	100.00	54.46	25.56	25.00	24.74	34.69	26.97	19.57	20.22	25.77	26.97	27.63	29.35	25.00	17.71	20.62
sp|Q5SY68|S1A7B_HUMAN	20.88	22.11	19.39	22.45	19.10	22.99	52.48	54.46	100.00	23.33	24.00	24.74	23.47	24.72	16.30	16.85	18.56	22.47	25.00	30.43	20.83	12.50	22.68
sp|P05109|S10A8_HUMAN	37.78	27.96	21.51	29.03	30.68	27.91	24.44	25.56	23.33	100.00	25.81	27.78	26.88	39.77	22.83	18.82	24.73	34.09	32.47	36.26	30.11	18.48	25.00
sp|P06702|S10A9_HUMAN	36.17	30.61	29.70	27.72	32.61	30.00	24.00	25.00	24.00	25.81	100.00	29.90	32.04	46.74	30.93	30.85	23.53	36.96	29.11	35.79	34.34	22.33	25.69
sp|P60903|S10AA_HUMAN	47.25	32.98	25.77	34.02	33.71	26.44	22.68	24.74	24.74	27.78	29.90	100.00	36.08	31.46	25.27	26.44	22.92	37.08	30.26	43.48	46.88	24.21	29.17
sp|P31949|S10AB_HUMAN	40.43	28.57	27.00	28.00	32.61	28.89	32.65	34.69	23.47	26.88	32.04	36.08	100.00	35.87	31.63	25.26	24.51	34.78	34.18	38.95	31.31	23.47	26.26
sp|P80511|S10AC_HUMAN	36.96	34.78	30.43	32.61	34.83	34.83	25.84	26.97	24.72	39.77	46.74	31.46	35.87	100.00	31.46	28.24	25.00	38.04	35.44	44.57	39.13	19.78	33.70
sp|Q99584|S10AD_HUMAN	34.07	29.47	26.60	29.79	36.67	29.89	19.57	19.57	16.30	22.83	30.93	25.27	31.63	31.46	100.00	37.36	28.87	25.84	32.91	26.09	26.60	15.05	26.88
sp|Q9HCY8|S10AE_HUMAN	24.42	23.86	18.68	25.27	21.43	25.00	19.10	20.22	16.85	18.82	30.85	26.44	25.26	28.24	37.36	100.00	21.74	27.06	21.79	23.53	19.10	21.11	16.67
sp|Q96FQ6|S10AG_HUMAN	32.98	29.59	23.23	31.31	29.35	27.78	25.77	25.77	18.56	24.73	23.53	22.92	24.51	25.00	28.87	21.74	100.00	28.26	30.38	27.37	29.29	21.65	24.49
sp|P04271|S100B_HUMAN	57.61	43.48	36.96	45.65	42.70	39.33	25.84	26.97	22.47	34.09	36.96	37.08	34.78	38.04	25.84	27.06	28.26	100.00	34.18	50.00	44.57	25.27	30.43
sp|P29377|S100G_HUMAN	40.51	35.44	25.32	36.71	35.06	35.06	28.95	27.63	25.00	32.47	29.11	30.26	34.18	35.44	32.91	21.79	30.38	34.18	100.00	34.18	37.97	24.05	32.91
sp|P25815|S100P_HUMAN	52.69	40.00	31.58	41.05	38.46	34.83	27.17	29.35	30.43	36.26	35.79	43.48	38.95	44.57	26.09	23.53	27.37	50.00	34.18	100.00	50.53	24.47	33.68
sp|Q8WXG8|S100Z_HUMAN	57.45	40.21	34.34	43.43	42.39	38.89	25.00	25.00	20.83	30.11	34.34	46.88	31.31	39.13	26.60	19.10	29.29	44.57	37.97	50.53	100.00	24.74	28.57
sp|Q5QJ38|TCHL1_HUMAN	23.91	18.95	22.22	20.20	22.22	22.73	16.67	17.71	12.50	18.48	22.33	24.21	23.47	19.78	15.05	21.11	21.65	25.27	24.05	24.47	24.74	100.00	20.92
sp|Q86YZ3|HORN_HUMAN	32.26	25.00	25.00	25.00	28.57	25.84	19.59	20.62	22.68	25.00	25.69	29.17	26.26	33.70	26.88	16.67	24.49	30.43	32.91	33.68	28.57	20.92	100.00
